# Persistent Organic Pollutant Exposure and Thyroid Function among 12-Year-Old Children

**DOI:** 10.1159/000528631

**Published:** 2022-12-09

**Authors:** Hélène Tillaut, Christine Monfort, Frank Giton, Charline Warembourg, Florence Rouget, Sylvaine Cordier, Fabrice Lainé, Eric Gaudreau, Ronan Garlantézec, Dave Saint-Amour, Cécile Chevrier

**Affiliations:** ^a^Univ Rennes, Inserm, EHESP, Irset (Institut de recherche en santé, environnement et travail) - UMR_S 1085, Rennes, France; ^b^AP-HP, Pôle Biologie-Pathologie Henri Mondor, Créteil, France; ^c^Inserm IMRB, Faculté de Santé, Créteil, France; ^d^Univ Rennes, CHU Rennes, Inserm, EHESP, Irset (Institut de recherche en santé, environnement et travail) - UMR_S 1085, Rennes, France; ^e^CHU Rennes, Inserm CIC1414, Rennes, France; ^f^Centre de Toxicologie du Québec (CTQ), Institut national de santé publique du Québec (INSPQ), Quebec, Québec, Canada; ^g^Département de Psychologie, Université du Québec à Montréal, Montréal, Québec, Canada; ^h^Centre de Recherche du Centre Hospitalier Universitaire Sainte-Justine, Montréal, Québec, Canada

**Keywords:** Persistent organic pollutant, Per- and polyfluoroalkyl substances, Thyroid function, Adolescence

## Abstract

**Introduction:**

Polychlorobiphenyls (PCBs), organochlorine pesticides (OCPs), and per- and polyfluoroalkyl substances (PFASs) are persistent organic pollutants (POPs) having numerous toxicological properties, including thyroid endocrine disruption. Our aim was to assess the impact of POPs on thyroid hormones among 12-year-old children, while taking puberty into consideration.

**Methods:**

Exposure to 7 PCBs, 4 OCPs, and 6 PFASs (in µg/L), and free tri-iodothyronine (fT3, pg/mL), free thyroxine (fT4, ng/dL), and thyroid-stimulating hormones (TSH, mIU/L) were assessed through blood-serum measurements at age 12 years in 249 boys and 227 girls of the PELAGIE mother-child cohort (France). Pubertal status was clinically rated using the Tanner stages. For each POP, associations were estimated using linear regression, adjusted for potential confounders.

**Results:**

Among boys, hexachlorobenzene and perfluorodecanoic acid were associated with decreased fT3 (log-scale; β [95% confidence interval] = −0.07 [−0.12,−0.02] and β = −0.03 [−0.06,−0.00], respectively). Intermediate levels of perfluorohexanesulfonic acid (PFHxS) and PCB180 were associated, respectively, with increased and decreased fT4. After stratification on pubertal status, PCBs and OCPs were associated with decreased TSH only in the more advanced Tanner stages (3–5) and with decreased fT3 among early Tanner stages (1–2). Among girls, PFHxS was associated with decreased TSH (log-scale; β = −0.15 [−0.29,-0.00]), and perfluorooctanoic acid was associated with decreased fT3 (β<sub>2nd_tercile</sub> = −0.06 [−0.10,-0.03] and β<sub>3rd_tercile</sub> = −0.04 [−0.08,-0.00], versus. 1st tercile).

**Discussion:**

This cross-sectional study highlights associations between some POPs and thyroid function disruption, which appears consistent with the literature. Considering that the associations were sex-specific and moderated by pubertal status in boys, complex endocrine interactions are likely involved.

## Introduction

Polychlorinated biphenyls (PCBs) and organochlorine pesticides (OCPs) are persistent organic pollutants (POPs) that are widespread in the environment. Because of their lipophilic properties, these chemicals bioaccumulate in both organisms (including humans) and food chains. Exposure occurs mainly via the consumption of contaminated food [1] but also via dust ingestion, absorption from dermal contact, and inhalation. The use of legacy POPs (PCBs and some OCPs) has been restricted by the UN Stockholm Convention since 2004, and other OCPs have been added to the list since then. Per- and polyfluoroalkyl substances (PFASs) are a group of synthetic chemicals having hydrophobic and lipophobic properties. They enter the composition of everyday consumer products, including food packaging, nonstick cookware, firefighting foam, and clothing [1]. Two of these, namely perfluorooctanoic acid (PFOA) and perfluorooctane sulfonic acid (PFOS), were added to the Stockholm Convention POP list. As a result of environmental contamination, the general population worldwide is widely exposed to POPs, which are a ubiquitous presence in human biological samples [2, 3].

Experimental in vivo and in vitro studies have shown that exposure to POPs leads to disruption of thyroid hormone (TH) homeostasis [4]. THs, tri-iodothyronine (T3) and thyroxine (T4), and thyroid-stimulating hormone (TSH) all play a major role in numerous human physiological processes − such as metabolism, cardiovascular functions, and growth and development in children. Small intrapersonal changes in thyroid function, within the normal reference range, have been linked to adverse health outcomes [5]. Furthermore, TH dysregulation or disease might also influence cognitive functions [6] and mental health, including dysregulated mood [7], depression [8, 9], behavior [10], and attention-deficit hyperactivity disorder in children [11, 12]. The most relevant mechanisms of thyroid dysfunction due to endocrine-disrupting chemicals are: disturbance of overall thyroid gland activity via interference with the TH or TSH receptors through stimulation, inhibition or interference with other receptors on the thyrocyte; stimulation or inhibition of the enzyme functions that mediate the thyroid gland iodine uptake in the synthesis of T3 and T4; and competitive displacement of THs on their binding protein [4].

Several epidemiological studies have highlighted associations between exposure to POPs and TH disruption in adults as well as in children and adolescents. Overall, though decreased TSH and increased THs were generally observed, results varied depending on the hormones and pollutants studied. Studies among children and young adults have reported either a drop in TSH serum level in relation to increasing serum PFOA in both sexes [13], or in girls [14], or a rise in TSH level with increasing hexachlorobenzene (HCB) [15], PCBs [16], PFOS, and perfluorononanoic acid (PFNA) in boys [14]. An increase in fT4 with increased PCBs, dichlorodiphenyldichloroethylene (p,p'-DDE), HCB [15], and PFOA [13], and with PFNA with sex-specific effects was also reported [13, 17, 18].

Pubertal development is a time windows that brings major hormonal modifications, including rises in sex hormones as well as changes to TH level [19, 20, 21], with overall decreases in TSH, free T3 (fT3), and free T4 (fT4) as well as transient peaks that can be specific to sex or pubertal stage [21]. In general, puberty involves complex neuroendocrine mechanisms that are influenced by several factors, including POPs. Several studies have investigated POP exposure in relation to pubertal timing, at either onset or late milestone points, such as age at menarche [22, 23, 24, 25, 26], but their results vary and remain inconclusive.

In the present study, we aimed to assess the impact of several POPs on THs (TSH, fT3, and fT4) levels measured in blood serum among a sample of French boys and girls aged 12 years. A secondary objective was to explore the associations between POP exposure and THs across pubertal status, as observed among the 12-year-old children.

## Materials and Methods

### Study Population

The PELAGIE mother-child cohort included 3,421 pregnant women from Brittany, France, between 2002 and 2006. Women were recruited before the 19th week of gestation by their gynecologist, obstetrician, or ultrasonographer at their first prenatal visit [27]. A total of 2,620 participants, not-lost-of-follow-up, were invited to the follow-up of the PELAGIE cohort organized when children reached 12 years of age. Of these, 1191 were eligible for the clinical examination (45%), which was restricted to those families for which a cord blood sample was collected at birth; 933 participants were contacted by phone and 559 agreed to their child's participation in a clinical examination conducted in a hospital setting. During this examination, puberty status was assessed by trained professionals, using Tanner's 5 stages [28], while age at menarche was self-reported. Children who were included in the clinical exam were drawn a 21 mL blood sample. The children's mothers completed a self-administered questionnaire on their family, social and demographic characteristics, diet, lifestyle, and the child's health. Adult participants provided written informed consent, and children provided a written assent.

### Thyroid Hormone Analyses

fT3, fT4, and TSH were measured in blood serum by immunoenzymatic colorimetric assay (DKO013 TSH ELISA Kit, DKO037 fT3 ELISA Kit, DKO038 fT4 ELISA Kit, DiaMetra). Analyses were performed at Henri Mondor Hospital in Paris. The results were expressed as concentrations in mIU/L (international unit) for TSH, pg/mL for fT3, and ng/dL for fT4. The intra- and interassay coefficients of variation were ≤4.6% and ≤10.8% for TSH, ≤4.94% and ≤13.19% for fT3, and ≤10.98% and ≤10.81% for fT4, respectively. The lowest detectable concentrations that can be distinguished from the calibrator 0 with a confidence limit of 95% were 0.01 mIU/L for TSH, 0.05 pg/mL for fT3, and 0.05 ng/dL for fT4. Serum concentrations of free T3, free T4, and TSH were used as main outcomes and we also explored the ratio of fT4/fT3 (using molar weights) as a possible marker of peripheral thyroid metabolism by deiodinase enzymes [29].

### Assessment of POP Exposure

Fourteen POPs were selected according to their potential of exposure of the general population in the Brittany coastal region (past usages, environmental contamination data, and human biomonitoring data) [30, 31]. For PFAS, selection was made on the basis of the national French studies on food contamination and biomonitoring [32]. Exposure to POPs was assessed by measuring biomarkers in blood serum samples (same sample as for TSH, fT3, and fT4 analysis): 7 PCBs (PCBs 118, 138, 153, 170, 180, 187, and 194), 4 OCPs (beta-hexachlorocyclohexane [beta-HCH], HCB, dieldrin, and p,p'-DDE), and 6 PFASs (PFOA, PFNA, perfluorodecanoic acid [PFDA], perfluoroundecanoic acid [PFUdA], perfluorohexane sulfonate [PFHxS], and PFOS) were determined by the Centre de toxicologie du Québec (CTQ) at the Institut national de santé publique du Québec (INSPQ).

Two milliliters of serum (or cord serum) samples were enriched, using internal labeled standards, and proteins were denaturized using reagent alcohol. The POP compounds (PCBs and OCPs) were extracted with hexane from the aqueous matrix using a liquid-liquid extraction in the presence of a saturated ammonium sulfate solution. These extracts were cleaned up on deactivated 0.5% florisil columns. Elution was broken down into 2 steps: the first fraction was eluted with a mixture of dichloromethane:hexane (25:75; 9 mL) and contains all compounds except heptachlor epoxide, endrin, dieldrin, endosulfan I, and endosulfan II, which were then eluted in the second fraction with a mixture of acetone (dichloromethane, 2:98, 4 mL). The solvent of the first fraction was evaporated, taken up in 125 μL of hexane concentrated to 20 μL, and analyzed for PCBs and OCPs on an Agilent 6890 Network or 7890A gas chromatograph equipped with an Agilent 7683 or 7693 series automatic injector and an Agilent 5973 Network or 5975C mass spectrometer (MS) (Agilent Technologies Inc.; Mississauga, ON, Canada). The GC was fitted with an Agilent 60 m DB-XLB column (0.25 mm i.d., 0.25 µm film thickness) to the MS and an Agilent Ultra-1 50 m (0.20 mm i.d., 0.33 µm film thickness) to the ECD. The carrier gas was helium, and the injections were 3 µL in the splitless mode. Fraction 2 was also evaporated, taken up in 20 μL of acetonitrile, and analyzed on a GC-MS described above. The injection was 2 µL in the splitless mode. All the MSs were operated in selected ion monitoring, using negative ion chemical ionization, with methane (99.97%) as the reagent gas. Total cholesterol (TC), free cholesterol (FC), triglycerides, and phospholipids levels were also measured in these samples by enzymatic methods (in g/L) and allowed to calculate the total lipid level as 1.677*(total cholesterol − FC) + FC + triglycerides + phospholipids [33].

The PFAS analysis was split into 2 sets. For the first set (boy samples), serum samples (100 μL) were enriched with labeled internal standards (PFBA-^13^C_4_, PFHxA-^13^C_6_, PFOA-^13^C_4_, PFNA-^13^C_9_, PFDA-^13^C_9_, PFUdA-^13^C_7_, PFHxS-^13^C_3_, and PFOS-^13^C_4_) and acidified with a 50% formic acid solution. Thereafter, the samples were extracted using a solid-phase extraction with a Strata-X AW 96-well plate 30 mg (33 µm) (Phenomenex; Torrance, CA, USA). After conditioning the 96-well plate with methanol and water and processing the samples, the resin was washed with a 2% formic acid solution, and methanol and analytes were eluted by the solution of 5% NH_4_OH in methanol. The extracts were evaporated to dryness and dissolved in 900 µL of 5 mM ammonium acetate in 40% methanol. The samples were analyzed using Ultra Performance Liquid Chromatography (UPLC Waters Acquity) with a tandem mass spectrometer (MS/MS Waters Xevo TQ-S) (Waters, Milford, MA, USA) in the MRM mode, with an electrospray ion source in the negative mode. The column used was an ACE EXCEL C18-PFP 50 mm × 2.1 mm, 2.0 µm (ACE; Aberdeen, Scotland). The mobile phase was consisted of a gradient of (30:70) methanol: H_2_O with 5 mM ammonium acetate to 100% methanol with 5 mM ammonium acetate in 14.6 min with a flow rate of 0.5 mL/min.

To improve the sensitivity and the precision of the method, the second set of PFAS analysis (girl samples) was done with essentially the same method than previous but with the following differences: the samples were extracted using a solid-phase extraction with SiliaPrep X WAX cartridges 100 mg/3 mL (SiliCycle; Québec, Canada). The extracts were reconstituted in 1 mL of 5 mM ammonium acetate in 20% acetonitrile. The column used for the analysis by UPLC-MS/MS was the same but longer: ACE EXCEL C18-PFP 100 mm × 2.1 mm, 2.0 µm. The mobile phase was consisted of a gradient of (10:90) acetonitrile: H_2_O with 5 mM ammonium acetate to 100% acetonitrile with 5 mM ammonium acetate in 7.0 min with a flow rate of 0.5 mL/min. Based on the Bland-Altman plots, the mean percentage differences between the two PFAS methods were lower than 3.2% for the analytes measured and calculated on the analysis of 66–150 samples depending on the level of detection of the analyte. The two methods were then considered equivalent. Blood samples of 476 of the 559 children who attended the clinical examination at the age of 12 years were analyzed for POP measurements. Concentrations were reported as wet weight (µg/L).

### Statistical Analyses

Following visual verification for normality distribution, TSH, fT3, and fT4 hormones were log-transformed. Pearson correlation coefficients between log-transformed concentrations were calculated. POP compounds detected in fewer than 70% of the samples were not included in the analyses. Distributions of selected POPs were graphically checked and concentrations were log-transformed. Values below the LOD were randomly imputed from a log-normal probability distribution, the parameters of which were estimated by a maximum-likelihood method [34]. Pearson correlation coefficients were calculated between imputed log-transformed POP concentrations.

#### Covariate Adjustment Strategy

Potential covariates were obtained from questionnaires completed at inclusion (first trimester of pregnancy), at birth or at the age of 2, 6, or 12 years. They were selected a priori on the basis of the existing literature on factors influencing THs. A minimal set of confounders were included, namely parental (maternal or paternal) history of thyroid hormonal disorders (yes/no), season, and time of day for the blood draw. For lipophilic POPs (PCBs and OCPs), total lipids were also included in the models. For additional potential confounders selection, passive tobacco smoke exposure (yes/no), number of years in full-time education for mother and father (in 3 groups: <12 years, 12 years, >12 years), and whether the child was breastfed (not at all, ≤3 months, >3 months) were also considered. For each hormone and each POP family (PCBs, OCPs and PFASs), we retained the covariates associated with the hormone and at least one POP using minimally adjusted models with *p* < 0.2 (except for PFOA that differed from other PFASs). Other than for breastfeeding history, selected covariates had very few missing data (<3.5%) and were then simply imputed with the mode. For breastfeeding (missing data *n* = 68, 16%), simple imputation was made with chained equations, using a proportional odds model for ordered variables [35] and 10 potential predictive variables, collected at inclusion and at birth. Among the predicted values, the proportions of children not breastfed and breastfed for less than 3 months or longer than 3 months were no different from those among observed values (pχ^2^ = 0.7).

#### Principal Analyses

We used separate linear regression models to estimate associations between each POP and each of the 3 hormones' serum concentrations (both log-transformed) and the fT4/fT3 ratio adjusted for the covariates selected in the minimal adjustment set. All these models were further adjusted for potential confounders (identified as listed above). Beta coefficients with 95% confidence intervals (CIs) were computed.

Linearity was investigated using models including restricted cubic splines adjusted for the minimal set of covariates. Models with exposure included as either a continuous variable or a restricted cubic spline were compared; linearity was rejected when the *p* value of the log-likelihood ratio test was <0.1 [36]. POP concentrations were then categorized into terciles in the regression models. All models were stratified on child sex.

#### Sensitivity Analyses

THs are involved in mechanisms that can affect BMI [37], but this relationship can also be reversed [38]. To avoid over adjustment in the main analysis, the potential influence of BMI was examined only in a sensitivity analysis by including child BMI as a continuous covariate in the fully adjusted models.

Considering that prenatal exposure to POPs can affect both maternal TH homeostasis and that of the child [39, 40, 41], the potential influence of POP concentrations in cord-serum samples was examined. At least one POP analysis in cord-serum sample was available for every child included in the main analysis (*n* = 476). The same analysis strategy (log-transformation, imputation, and linearity testing) as for POPs analyzed at the age of 12 years was used. Spearman rank correlations between prenatal and 12-year POP concentrations were less than 0.3. We then included in each model the corresponding cord-serum log-transformed POP concentration (as well as total lipid concentration in cord serum for PCBs and OCPs) to check whether the results were affected. For this sensitivity analysis, both the main models and those models further adjusted for cord-serum POP concentrations were run on the samples of children having data both for cord serum and 12-year-olds' serum for each POP.

#### Secondary Analysis

Pubertal status was categorized into 3 groups for boys and girls: (1) Tanner stages 1 and 2, representing a somewhat delayed puberty; (2) Tanner stage 3, expected at this age; and (3) Tanner stages 4 and 5, advanced puberty. Linear regression models were fitted, first including an interaction term between exposure and pubertal status, and then stratifying on these 3 groups for boys and girls separately. The minimal set of covariates identified in the principal analysis was used for adjustment.

All analyses were performed using R software [42]. A *p* value of <0.05 was considered to be statistically significant, and the heterogeneity effect was reported when the *p* value for interaction was <0.2.

## Results

Measurements for THs, and at least one POP were available for 476 children (249 boys and 227 girls). The characteristics of the study population are summarized in Table 1. The mean age at clinical examination was 12.8 years (SD = 0.1). More than 90% of children came from families with adults living in couples, and more than 70% of mothers and 60% of fathers were highly educated (more than 12 years of studies). Around 30% of children were exposed to passive tobacco smoking (i.e., at least one adult smoking at home). Thirteen percent of mothers and 1% of fathers reported having been diagnosed with thyroid disorders. A little less than half of girls (41.4%) were postmenarche, and 8% of boys were considered prepubertal (Tanner stage 1).

TH distributions are summarized in Table 2. Median TSH was 0.87 mIU/L for boys and girls (range: 0.23–5.76 and 0.21–3.65, respectively). Median fT3 was 3.32 pg/mL (range: 2.49–4.40) for boys and 3.25 pg/mL (range: 2.42–6.41) for girls. Median fT4 was 0.98 ng/dL (range: 0.55–1.48) for boys and 0.95 ng/dL (range: 0.51–1.34) for girls. Correlation coefficients reached 0.25 between log-transformed fT3 and TSH, and 0.31 between fT3 and fT4 for girls (*p* < 0.001) and 0.17 between log-transformed fT3 and fT4 (*p* < 0.01) for boys.

Concentrations of TSH did not differ according to pubertal status among boys or girls. fT3 concentrations increased in boys across puberty stages 1–2, 3, and 4–5, from 3.21 pg/mL to 3.42 pg/mL (geometric mean) and decreased in girls from 3.32 pg/mL to 3.14 pg/mL. fT4 concentrations decreased among boys from 1.03 ng/dL (Tanner stages 1–2) to 0.94 ng/dL (Tanner stages 4–5) and did not differ according to the pubertal status among girls (Table 3).

Table 4 presents the distribution of POP serum concentrations for boys and girls. PCB 138, 153, 180, HCB, p,p'-DDE, PFOA, PFNA, PFDA, PFUdA, PFHxS, and PFOS were detected in more than 90% of samples, followed by beta-HCH detected in 91% of samples among boys and 80% among girls, and PCB 118 detected in 75% of samples for both sexes.

Overall, median concentrations were higher for boys than for girls. The POP Pearson correlation coefficients are shown in the supplemental material (online suppl. Fig. S1; for all online suppl. material, see www.karger.com/doi/10.1159/000528631). For both sexes, the strongest correlations were between PCB 138, 153, and 180 (*r* ≥ 0.9). Of the OCPs, correlation coefficients reached 0.5 for girls and 0.4 for boys. For the PFAS family, the highest coefficients were observed between PFDA and PFUdA (*r* = 0.8) and PFOS (*r* = 0.7) for girls, and, between PFDA and PFUdA and PFOA (*r* = 0.6) and PFNA (*r* = 0.5) for boys. Between families, higher correlation coefficients were observed between p,p'-DDE and beta-HCH and PCB 138, 153, and 180 (*r* values ≥0.7 for girls and boys).

### Associations between POP Serum Concentrations and Thyroid Hormones and TSH Serum Concentrations

#### Boys

Overall, POP exposure was associated with decreased TSH, fT3, and fT4, with the exception of PFAS exposure, showing null association with fT4. In particular, HCB and PFDA serum concentrations were associated with decreasing fT3 serum concentration (β = −0.07 95% CI = [−0.12,−0.02] and β = −0.03 95% CI = [−0.06,−0.00], respectively). PCB 180 was associated with decreased fT4 in a nonlinear way, with a higher beta coefficient for the 2nd tercile than for the 3rd tercile (β_2nd vs 1st tercile_ = −0.04 95% CI = [−0.08,−0.00] and β_3rd vs 1st tercile_ = 0.00 95% CI = [−0.04, 0.04]) and PFHxS was associated with increased fT4, with higher beta coefficients for the 2nd tercile than for the 3rd tercile (β_2nd vs. 1st tercile_ = 0.04 95% CI = [0.00,0.08] and β_3rd vs. 1st tercile_ = 0.02 95% CI = [−0.02,0.06], Table 5). Similar results were obtained when we adjusted models for the minimal set of confounders (see online suppl. Table S1, shown in the supplemental material). Results for the fT4/fT3 ratio were mainly driven by fT3 results and showed similar conclusions, except that association with PFNA was stronger, and statistically significant (see online suppl. Table S2 in supplemental material). The shape of the statistically significant associations, using restricted cubic splines, is shown in online supplementary Figure S2 in supplemental material.

#### Girls

For girls, POP exposure, overall, was associated with decreased TSH and fT3 (Table 6). In particular, PFHxS serum concentration was associated with decreased TSH concentrations (β = −0.15 95% CI = [−0.29,−0.00]) and PFOA was associated with decreased fT3 (β_2nd vs 1st tercile_ = −0.06 95% CI = [−0.10,-0.03] and β_3rd vs 1st tercile_ = −0.04 95% CI = [−0.08,−0.00]). Similar results were obtained when adjusting for the minimal set of confounders, though the association between HCB and increased fT4 was significant (see online suppl. Table S1 in supplemental material). Results for the fT4/fT3 ratio showed similar conclusions than those for fT3 (see online suppl. Table S2 in supplemental material).

### Sensitivity Analysis

#### Boys

When considering further adjustment for cord-serum POP concentrations (see online suppl. Table S3 in supplemental materials for the cord-serum POP distributions), the negative associations between beta-HCH and TSH were strengthened. The other associations were not modified (see online suppl. Table S4). When further adjusted on BMI, results remained globally unchanged, except for the association between HCB and decreased fT3 that was no longer statistically significant (see online suppl Table S6).

#### Girls

When considering further adjustment for cord-serum POP concentrations, relationships remained globally unchanged. When further adjusted for BMI, the associations between PCB exposure and TSH appeared reversed though without statistical significance, and the association between beta-HCH and increased TSH became statistically significant. Other results remained globally unchanged (see supplemental material for the results of models further adjusted for cord-serum POP concentrations and BMI, online suppl. Tables S5, S6).

### Secondary Analysis − Associations between POP Serum Concentrations and Thyroid Hormones and TSH Serum Concentrations Stratified on Pubertal Status

#### Boys

Suggestive evidence for a heterogeneous effect across pubertal status was observed for the associations between exposure to PCB and TSH and between exposure to PCB (118 and 138) and p,p'-DDE and fT3 (*p* value for interaction <0.2, Tables 7, 8). After stratification on pubertal status, PCB and OCP exposures were associated on the whole with decreased TSH among boys having reached Tanner stages 3 and 4–5 (Table 7). The association between HCB and decreased TSH was statistically significant for Tanner stages 4–5 (β = −0.56 95% CI = [−1.10,−0.02]). Statistical significance was almost reached for Tanner stages 3 and 4–5 for PCB 138, and for Tanner stage 3 for PCB 153, p,p'-DDE and beta-HCH. For PFAS, no clear pattern was observed, other than for PFOS which was associated with decreased TSH (Tanner stages 4–5: β = −0.25 95% CI = [−0.48,−0.02]). For fT3 (Table 8), the association between PCB and OCP exposure and decreased fT3 were stronger among early pubertal boys (Tanner stages 1–2): in particular, PCB 118 (β_2nd vs 1st tercile_ = −0.01 95% CI = [−0.08,0.06] and β_3rd vs 1st tercile_ = −0.07 95% CI = [−0.14,−0.00]), p,p'-DDE (β_2nd vs 1st tercile_ = −0.00 95% CI = [−0.07,0.06] and β_3rd vs 1st tercile_ = −0.09 95% CI = [−0.17,−0.01]). PFOA exposure was associated with increased fT3 in early pubertal boys (Tanner stages 1–2: β = 0.09 95% CI = [0.00,0.18]). No clear heterogeneous pattern was observed regarding the associations between POP exposure and fT4 among boys, across pubertal status (Table 8).

#### Girls

No clear evidence of effect heterogeneity was observed among girls (see online suppl. Tables S7.1, S7.2 in supplemental material), although the association between p,p'-DDE and decreased TSH became statistically significant for the 3rd versus the 1st tercile of exposure (β_2nd vs 1st tercile_ = −0.10 95% CI = [−0.31,0.12] and β_3rd vs. 1st tercile_ = −0.25 95% CI = [−0.47,−0.03]) among girls in Tanner stage 3.

## Discussion

The aim of this study was to assess the impact of POP exposure on thyroid function among adolescents at the age of 12 years by examining TSH, fT3, and fT4. Overall, we observed a decrease of TSH, fT3, and fT4 with the exposure levels of several PCBs and OCPs, generally more accentuated among boys. In our secondary analysis, we observed that associations between POPs and THs differ according to the pubertal status among boys. Particularly, we found more pronounced associations between PCBs and OCPs and decreased TSH in boys having reached Tanner stage 3 and above. We also found that associations between PCBs and OCPs and decreased fT3 were higher among boys in early puberty (Tanner stages 1–2).

Human studies have shown PCBs to be associated with decreased levels of THs (fT3 and fT4) levels or increased TSH levels [43]. As expected, we did indeed find overall negative associations between PCBs and fT3 and fT4 among both boys and girls, though few of these reached statistical significance. Overall, we found fairly negative or null associations between PCB exposure and TSH levels among both boys and girls; however, these associations were stronger among those boys who were advanced in terms of puberty (Tanner stages 3 and above). The link between PCBs and THs has been highlighted in the literature: Osius et al. [16] identified an association between PCB 118 and increased TSH among children aged 7–10 years as well as an association between PCBs 138, 153, 180, 183, and 187 and decreased fT3, while Croes et al. [15] reported positive correlations between PCB 138, 153, and 180 and fT4 in Belgian adolescents.

For OCPs, as reviewed by Langer [44] and Leemans et al. [45], the evidence of TH-disrupting effects of OCPs (such as p,p'-DDE, beta-HCH, and HCB) is weaker than for PCBs, at least insofar as adults are concerned. A small number of human studies on pesticide exposure have found HCB to be associated with decreased TH levels [44, 45]. Numerous animal studies have shown exposure to pesticides to be associated with decreased TH levels [43]. Among children, Freire et al. [46] identified a significant and rising linear trend between HCB, beta-HCH, and p,p'-DDE and fT3 in Brazilian children aged 6 years. Croes et al. [15] identified positive correlations between p,p'-DDE and HCB and fT4 levels in Belgian adolescents; they also found increased TSH to be associated with HCB. Conversely, in our study, we observed overall associations between OCPs and decreased TSH and fT3 among boys and girls, with the exception of beta-HCH and TSH among girls. The associations between OCPs and TSH were stronger among boys having reached Tanner stage 3 and above, while the association between p,p'-DDE and fT3 was stronger among early pubertal boys. Leemans et al. [45] concluded, based on epidemiologic, in vivo and in vitro studies, that the underlying mechanisms are multiple and complex, including mimicking TH properties, binding to thyroid receptors or transport proteins, fastening TH clearance, increased hepatic metabolism, and indirect effects (such as on growth factor, which can in turn impact thyroid function).

Concerning the PFAS family, a review by Lee et Choi [47] concluded, in view of experimental and (to a lesser extent) human studies, that PFASs were associated with a decrease in THs (T3 and T4) and an increase in TSH. In adults, a recent review by Coperchini et al. [48] reported that PFAS associations with THs were sex-specific, with positive and negative associations depending on PFAS types and hormones. In our study in early adolescents, the associations between PFASs and THs were also sex-specific (PFHxS and fT4 and PFDA and fT3 among boys vs. PFHxS and TSH and PFOA and fT3 among girls), although they seemed less prone to effect modifications of pubertal status. Lewis et al. [14] identified that during adolescence (American adolescents aged 12–20 years), both PFOS and PFNA were associated with increased TSH in males, whereas PFOA was inversely related to TSH in females. Caron-Beaudoin et al. [17] found PFNA (median concentration 1.18 µg/L, which is higher than the median in the present study) to be associated with increased T4 in Canadian boys and girls. Finally, Lin et al. [18] also found PFNA (median concentration 1.01 ng/mL) to be associated with increased fT4, especially among men aged 20–30 years. As reported by several in vitro studies [48], the action mechanisms of PFAS endocrine disruption may involve cytotoxicity, genotoxicity, interferences with TH synthesis, TPO function, and iodine uptake.

The blood samples collected in our study when children were 12 years old reflect chronic exposure to POPs. Gallo et al. [49] estimated that the half-lives of PCB 118, 138, and 153 range between 3 and 22 years, p,p'-DDE between 7 and 35 years, and HCB between 9 and 70 years. Median concentrations of PCBs were lower in our study than those described by Osius et al. in 1999 [16] and Schell et al. in 2008 [50]; yet were similar to those reported by Bandow et al. [51] in Germany for children aged 3–17 years between 2014 and 2017 (PCB 138: 0.02 µg/L, PCB 153: 0.07 µg/L; and PCB 180: 0.03 µg/L). The PFASs analyzed in our study were long-chain PFASs with half-lives in humans ranging from 3½, 5 years for PFOA to 10 years for PFHxS [47]. Median concentrations in our study were similar to those obtained from children aged 6–17 years in France [2]: PFOA: 1.54 µg/L serum, PFNA: 0.57 µg/L, PFDA: 0.24 µg/L, PFHxS: 0.73 µg/L, and PFOS: 2.00 µg/L. In our study, boys had higher POP serum concentrations than girls. Among children aged 3–17 years, Bandow et al. also showed higher concentrations of PCB 138, 153, 180 and HCB in boys' serum; this difference could be due to differences in the proportion of adipose tissue between the sexes [51], which was higher among girls in our study. Due to the POPs' long half-lives, the period when modification in thyroid function might have occurred through POPs endocrine disruption effect is, therefore, unidentified and might be long before the study took place.

A complex relationship occurs between circulating TSH and T4 levels: minor changes in T4 can lead to major variations in TSH levels. Thyroid function varies across ages and TSH, fT3, and fT4 levels present a wider range in children than in adults. Furthermore, puberty may have some impact on the pituitary-thyroid axis function. Surup et al. [21] have shown that a decrease in TSH occurs during puberty, alongside a decrease in fT3 with a temporary peak in males, and a temporary nadir of fT4 in Tanner stage 3 for both sexes. Marwaha et al. [52] have shown that fT3 levels increase, while children of both sexes enter puberty, then either decrease or remain stable at adolescence. The authors also showed a decrease in fT4 levels and TSH levels, the latter decreasing in boys only. In Australian children, Campbell et al. [19] highlighted an increase in fT4 among girls only and more of an increase in fT3 in boys than girls, as well as no change in TSH between the ages of 12 and 14 years. These changes may reflect complex relationships between the growth hormone, hypothalamo-pituitary-gonadal, and hypothalamo-pituitary-thyroid axis [19, 52].

Exposure to POPs during pregnancy has been shown to alter thyroid function in pregnant women [39, 53, 54, 55], and to a lesser extent or with inconclusive results in newborns and infants [39, 41, 54, 56]. These alterations may impair the child's future health [4, 43], including their neuropsychological development [57]. The association of prenatal exposure to POPs and thyroid function among adolescents was investigated by Schell et al. [50] by using breastfeeding as a proxy. They found that persistent PCB serum concentration measured at adolescence (mean age 13.3 +/− 1.9 years) was positively associated with TSH levels in individuals who had not been breastfed − despite the fact that breastfed adolescents showed higher PCB levels. The authors interpreted this finding as a predominant prenatal origin of exposure in nonbreastfed children, which is thought to be more harmful than postnatal exposure. In the present study, the associations were not modified when adjusted on prenatal exposure, though they were slightly reinforced for TSH.

We found that the relationships between POP exposure and THs might be modified according to pubertal stage, among boys in particular. This is in line with Fudvoye et al., [58], pointing out that estimation of POP endocrine disruption effects on puberty is complex due to its effects on endocrine control of maturation of hypothalamic-pituitary development, including thyroid function. POP exposure has also been shown to influence sex hormones in adolescents [59, 60], suggesting that their impact on thyroid function might be direct, and indirect through gonadal hormone influences. The interactions between gonadal and THs are well established in experimental studies. For instance, estrogen has been shown to have various effects on thyroid function, such as increasing the thyroxine-binding globulin, which is the main transport protein for THs in the circulation [61]. The onset of puberty in males begins between 9 and 14 years old, but in females, puberty generally starts earlier, between 8 and 13 years old [28]. Thus, the mean age of participants in our study (12.8 years old) was perhaps not optimal (i.e., too late) to study the influence of puberty among girls. Because of our limited sample size, we were unable to study the associations between POP exposure and each Tanner stage − and it might have led to a lack of statistical power. We chose to categorize our sample by grouping Tanner stages 1 and 2 together to represent somewhat delayed puberty. The Tanner stage 3 group can be considered the expected puberty status for this age group, and Tanner stages 4 and 5 were also grouped together to represent adolescents with advanced puberty for their age group. Due to the putative effects of POP exposure on puberty onset [22, 23, 24, 25, 26], we cannot rule out the fact that our study participants might have been misclassified regarding their pubertal status. We also adjusted for potential covariates, yet the fact that the associations we observed could be due to residual confounding cannot be ignored.

Lastly, while the present study suggests that among boys, sensitivity to POP exposure effects on thyroid function might differ according to their pubertal development, these results must be confirmed by further studies. New generation compounds of PFAS (such as GenX, perfluorobutane sulfonic acid, and C604) must also be investigated as they are now spreading worldwide in replacement of PFOA and PFOS, which have been added to the Stockholm Convention list of banned or restricted POPs.

Consistent with the literature, our study has highlighted associations between several POPs and thyroid function within the pubertal development during which complex hormonal changes occur. This is important as thyroid function disruption in early adolescence can have adverse effects at physiological, cognitive, and mental health levels. These results invite further investigation of the question in other studies featuring similar background-level POP exposure, as endocrine disruption may still be a matter of concern.

## Statement of Ethics

The adults participating in this study provided their written informed consent and the children provided a written assent. Written informed consent was obtained from parents/legal guardians for all participants aged under 18 years. The Advisory Committee on Information Processing in Health Research (CCTIRS; 2015; no. 15.326bis), the Committee for the Protection of Persons (CPP; 2015; no. 15/23-985), and the French National Commission for Information Technology and Civil Liberties (CNIL; 2002, 2015; no. 915420/2015-456) approved this study.

## Conflict of Interest Statement

The authors have no conflicts of interest to declare.

## Funding Sources

The PELAGIE cohort has been funded by Inserm (since the beginning), the French Ministries of Health (2003–2004), Labor (2002–2003), and Research (ATC 2003–2004), the French National Institute for Public Health Surveillance (InVS, 2002–2006), the National Agency for Research (ANR, 2005–2008, 2010–2012, 2015–2019), the French Agency for Environmental Health Safety (Afsset/ANSES, 2007–2009, 2009–2012), the French Agency for Drug Safety (2013–2017), the Fondation de France (2014–2017, 2015–2018, 2017–2021), the French Ministry of Ecology (PNRPE 2014–2016), the Research Institute of Public Health (IResP 2011–2014), and the following European programs: Hi-WATE 2007–2009, ENRIECO 2008–2010, and OBERON 2019–2023. This research is part of a PhD project funded by the French network of doctoral programs, coordinated by EHESP French School of Public Health.

## Author Contributions

Substantial contributions to the conception or design of the work (Hélène Tillaut, Dave Saint-Amour, Cécile Chevrier, Sylvaine Cordier, Ronan Garlantézec, Florence Rouget); or the acquisition (Eric Gaudreau, Franck Giton, Christine Montfort, Florence Rouget, Fabrice Lainé), analysis (Hélène Tillaut, Charline Warembourg, Dave Saint-Amour, Cécile Chevrier), or interpretation of data for the work (Hélène Tillaut, Dave Saint-Amour, Cécile Chevrier, Charline Warembourg, Florence Rouget); drafting the work (Hélène Tillaut, Dave Saint-Amour, Cécile Chevrier) or revising it critically for important intellectual content (all); final approval of the version to be published (all); and agreement to be accountable for all aspects of the work in ensuring that questions related to the accuracy or integrity of any part of the work are appropriately investigated and resolved (all).

## Data Availability Statement

The PELAGIE cohort data comply with the European regulation on the protection of personal data (May 25, 2018). This regulation is based on a logic of compliance and increased responsibility of the actors who access to the data. In addition, the cohort study complies with the French “informatique et liberté” law (law *n*°78-17, January 1978, 2018). Access to data is thus possible after the agreement of the cohort principal investigators (Cécile Chevrier, Charline Warembourg) and if the actors demonstrate respect for these European and French principles of personal data protection to strengthen the rights of individuals. Further enquiries can be directed to the corresponding author.

## Supplementary Material

Supplementary data

## Figures and Tables

**Table 1:** Characteristics of the study population for boys (*n* = 249) and girls (*n* = 227)

Characteristics	Boys		Girls	
	*N*	%	*N*	%
Age of child, years				
mean (SD)	12.81 (0.14)		12.81 (0.13)	
NA	0		0	
Marital status				
couple	231	92.8	208	92.4
separated	18	7.2	17	7.6
NA	0		2	
BMI				
mean (SD) kg/m^2^	17.86 (2.20)		18.47 (2.72)	
NA	0		0	
Socioeconomic status				
Mother's education				
<12 years	21	8.5	18	8.1
12 years	32	13.0	43	19.4
≥12 years	194	78.5	161	72.5
NA	2		5	
Father's education				
<12 years	44	18.1	47	21.7
12 years	47	19.3	39	18.0
≥12 years	152	62.6	131	60.4
NA	6		10	
Passive tobacco smoking				
Non exposed	182	73.4	151	68.6
Exposed	66	26.6	69	31.4
NA	1	0.4	7	3.1
Thyroid hormonal disorders among parents			
Mother				
No	211	85.8	191	87.2
Yes	35	14.2	28	12.8
NA	3		8	
Father				
No	199	98.0	197	99.5
Yes	4	2.0	1	0.5
NA	46		29	
Child breastfed				
No	64	29.8	54	28.0
≤3 months	60	27.9	53	27.5
>3 months	91	42.3	86	44.6
NA	34		34	
Puberty	Voice changed (boys)		Menarche (girls)	
No	202	82.1	133	58.6
Yes	44	17.9	94	41.4
NA	3		0	
Tanner stages	Male external genitalia		Female breast development
Stage 1	20	9.0	8	3.5
Stage 2	54	24.2	46	20.3
Stage 3	87	39.0	122	53.7
Stage 4	50	22.4	42	18.5
Stage 5	12	5.4	9	4.0
NA	26		0	
Timing of blood draw				
Season				
Spring	72	28.9	61	26.9
Summer	66	26.5	61	26.9
Autumn	55	22.1	66	29.1
Winter	56	22.5	39	17.2
NA	0		0	
Day time				
Mean (SD)	13h 32 (2h 14)		13h 42 (2h 13)	
Morning	92	36.9	80	35.2
Afternoon	157	63.1	147	64.8
NA	0		0	

Female breast development scale: Stage 1: No glandular breast tissue palpable. Stage 2: Breast bud palpable under the areola (first pubertal sign in females). Stage 3: Breast tissue palpable outside areola; no areolar development. Stage 4: Areola elevated above the contour of the breast, forming a “double scoop” appearance. Stage 5: Areolar mound recedes into single breast contour with areolar hyperpigmentation, papillae development, and nipple protrusion. Male external genitalia scale: Stage 1: Testicular volume <4 mL or long axis <2.5 cm. Stage 2: 4-8 mL (or 2.5 to 3.3 cm long), 1st pubertal sign in males. Stage 3: 9-12 mL (or 3.4 to 4.0 cm long). Stage 4: 15-20 mL (or 4.1 to 4.5 cm long). Stage 5: >20 mL (or >4.5 cm long).

**Table 2:** Distribution of TSH, fT3, and fT4 hormones among boys and girls aged 12 years

Percentiles	TSH		fT3				fT4			
	Boys (*n* = 249)	Girls (*n =* 227)	Boys (*n* = 249)		Girls (*n =* 227)		Boys (*n* = 249)		Girls (*n =* 227)	
	mlU/L	mlU/L	pg/mL	pmol/L^a^	pg/mL	pmol/L^a^	ng/dL	pmol/L^b^	ng/dL	pmol/L^b^
2.5	0.38	0.35	2.67	4.11	2.61	4.01	0.78	10.04	0.78	10.04
10	0.51	0.49	2.90	4.45	2.80	4.30	0.87	11.20	0.86	11.07
25	0.66	0.65	3.09	4.75	2.99	4.59	0.92	11.84	0.91	11.71
50	0.87	0.87	3.32	5.10	3.25	4.99	0.98	12.61	0.95	12.23
75	1.27	1.23	3.56	5.47	3.48	5.34	1.04	13.39	1.03	13.19
90	1.65	1.59	3.76	5.78	3.71	5.70	1.12	14.42	1.09	14.08
97.5	2.14	2.28	4.01	6.17	4.06	6.23	1.25	16.09	1.20	15.45
Geometric mean (SD)	0.90 (1.61)	0.88 (1.63)	3.31 (1.10)		3.24 (1.13)		0.98 (1.13)		0.96 (1.12)	

TSH, thyroid-stimulating hormone; fT3, free tri-iodothyronine; fT4, free thyroxine.

a T3 PubChem molar weight 650.97 g/mol.

b T4 PubChem molar weight 776.87 g/mol.

**Table 3:** TSH, fT3, and fT4 levels according to pubertal status, among boys and girls aged 12 years

Geometric mean (SD)	*N*	TSH (mlU/L)	fT3 (pg/mL)	fT4 (ng/dL)
Boys	223			
Tanner stages 1-2 (genitalia)	74	0.90 (1.64)	3.21 (1.13)	1.03 (1.13)
Tanner stage 3 (genitalia)	87	0.91 (1.58)	3.36 (1.10)	0.97 (1.11)
Tanner stages 4-5 (genitalia)	62	0.89 (1.71)	3.42 (1.09)	0.94 (1.15)
*p* (ANOVA)		*0.9*	*0.0009*	*0.0004*
Girls	227			
Tanner stages 1-2 (breast)	54	0.86 (1.70)	3.32 (1.12)	0.96 (1.15)
Tanner stage 3 (breast)	122	0.86 (1.66)	3.25 (1.13)	0.96 (1.11)
Tanner stages 4-5 (breast)	51	0.97 (1.48)	3.14 (1.14)	0.96 (1.11)
*p* (ANOVA)		*0.3*	*0.05*	*0.9*

TSH, thyroid-stimulating hormone; fT3, free tri-iodothyronine; fT4, free thyroxine; SD, standard deviation.

**Table 4:** POP serum concentrations, among boys and girls aged 12 years

POP	Sex	LOD (µg/L)	*N*	% ND	Q10 (µg/L)	Q25 (µg/L)	Q50 (µg/L)	Q75 (µg/L)	Q90 (µg/L)
PCB118	boys	0.01	249	23.69	<0.01	0.010	0.014	0.020	0.029
	girls	0.01	227	27.75	<0.01	<0.01	0.013	0.020	0.031
PCB138	boys	0.01	249	0.40	0.017	0.022	0.034	0.048	0.066
	girls	0.01	227	3.52	0.013	0.019	0.026	0.041	0.072
PCB153	boys	0.01	249	0.00	0.030	0.042	0.065	0.100	0.132
	girls	0.01	227	0.00	0.023	0.033	0.052	0.084	0.144
PCB180	boys	0.01	249	1.61	0.014	0.020	0.037	0.063	0.100
	girls	0.01	227	9.69	0.010	0.016	0.028	0.055	0.092
HCB	boys	0.02	249	0.00	0.037	0.043	0.054	0.065	0.075
	girls	0.02	227	0.44	0.032	0.037	0.045	0.055	0.068
p,p'-DDE	boys	0.02	249	0.00	0.060	0.070	0.092	0.140	0.192
	girls	0.02	227	0.00	0.050	0.060	0.087	0.130	0.190
Beta-HCH	boys	0.01	248	8.87	0.010	0.010	0.020	0.023	0.033
	girls	0.01	227	20.26	<0.01	0.010	0.010	0.020	0.031
PFOA	boys	0.07	249	0.00	0.898	1.100	1.400	1.600	1.920
	girls	0.02	227	0.00	0.816	0.970	1.200	1.400	1.700
PFNA	boys	0.09	231	0.00	0.300	0.400	0.480	0.610	0.740
	girls	0.01	227	0.00	0.300	0.360	0.440	0.560	0.838
PFUdA	boys	0.05	231	11.26	<0.05	0.070	0.100	0.100	0.200
	girls	0.01	227	0.00	0.064	0.080	0.100	0.140	0.180
PFDA	boys	0.06	234	0.00	0.100	0.200	0.200	0.260	0.327
	girls	0.01	227	0.00	0.120	0.150	0.180	0.220	0.280
PFHxS	boys	0.06	249	0.00	0.438	0.550	0.690	0.890	1.200
	girls	0.03	227	0.00	0.396	0.480	0.580	0.780	1.100
PFOS	boys	0.43	249	0.00	1.700	2.100	2.800	3.800	5.200
	girls	0.07	227	0.00	1.700	2.000	2.500	3.100	4.500

LOD, limit of detection; ND, not detected; HCB, hexachlorobenzene; p,p'-DDE, dichlorodiphenyldichloroethylene; beta-HCH, beta-hexachlorocyclohexane; PFOA, perfluorooctanoic acid; PFNA, perfluorononanoic acid; PFDA, perfluorodecanoic acid; PFUdA, perfluoroundecanoic acid; PFHxS, perfluorohexane sulfonate; PFOS, perfluorooctane sulfonate.

**Table 5:** Associations between POP serum concentrations and TSH, fT3, and fT4 serum concentrations at age 12 years among boys (*n* = 249)

	TSH (mlU log-transformed)		fT3 (pg/mL log-transformed)		fT4 (ng/dL log-transformed)	
Exposure (µg/L log-transformed)	ß	95% Cl	ß	95% Cl	ß	95% Cl
PCB118	−0.068	(−0.192, 0.057)^a^			−0.015	(−0.048, 0.019)^a^
PCB118 (T2 vs. T1)			−0.029	(−0.062, 0.004)^b^, ^#^		
PCB118 (T3 vs. T1)			−0.028	(−0.059, 0.004)^b^, ^#^		
PCB138	−0.072	(−0.188, 0.045)^a^	−0.015	(−0.041, 0.011)^b^	−0.008	(−0.039, 0.023)^a^
PCB153	−0.047	(−0.149, 0.055)^a^			−0.004	(−0.031, 0.023)^a^
PCB153 (T2 vs. T1)			−0.025	(−0.057, 0.007)^b^		
PCB153 (T3 vs. T1)			−0.013	(−0.046, 0.019)^b^		
PCB180	−0.022	(−0.103, 0.059)^a^				
PCB180 (T2 vs. T1)			−0.028	(−0.060, 0.004)^b^, ^#^	−0.041	(−0.080, −0.002)^a^, *
PCB180 (T3 vs. T1)			−0.025	(−0.059, 0.008)^b^	−0.004	(−0.044, 0.036)
HCB	−0.149	(−0.378, 0.080)^a^	−0.072	(−0.122, −0.022)^c^, **	−0.031	(−0.092, 0.030)
p,p'-DDE					0.003	(−0.026, 0.033)
p,p'-DDE (T2 vs. T1)	−0.015	(−0.159, 0.130)^a^	0.008	(−0.023, 0.040)^c^		
p,p'-DDE (T3 vs. T1)	−0.068	(−0.225, 0.090)^a^	−0.015	(−0.050, 0.019)^c^		
Beta-HCH	−0.087	(−0.189, 0.016)^a^, ^#^	−0.009	(−0.032, 0.013)^c^		
Beta-HCH (T2 vs. T1)					−0.007	(−0.044, 0.031)
Beta-HCH (T3 vs. T1)					0.023	(−0.018, 0.064)^a^
PFOA	−0.002	(−0.178, 0.174)	−0.017	(−0.056, 0.021)^d^		
PFOA (T2 vs. T1)					0.012	(−0.026, 0.050)
PFOA (T3 vs. T1)					0.021	(−0.019, 0.060)
PFNA	0.072	(−0.091, 0.234)	−0.009	(−0.046, 0.028)^d^	0.022	(−0.024, 0.068)
PFUdA	−0.041	(−0.153, 0.072)			0.000	(−0.029, 0.030)
PFUdA (T2 vs. T1)			0.005	(−0.026, 0.037)^d^		
PFUdA (T3 vs. T1)			−0.010	(−0.049, 0.030)^d^		
PFDA	−0.108	(−0.242, 0.027)	−0.035	(−0.064, −0.005)^d^, *	0.012	(−0.025, 0.048)
PFHxS	0.056	(−0.087, 0.198)	−0.013	(−0.044, 0.019)^d^		
PFHxS (T2 vs. T1)					0.039	(0.000, 0.078)*
PFHxS (T3 vs. T1)					0.022	(−0.017, 0.061)
PFOS	−0.047	(−0.167, 0.072)	−0.016	(−0.043, 0.010)^d^	0.002	(−0.030, 0.033)

All models adjusted for parental history of thyroid disease, season, and hour of blood drawing. HCB, hexachlorbenzene; p,p'-DDE, dichlorodiphenyldichloroethylene; beta-HCH, beta-hexachlorocyclohexane; PFOA, perfluorooctanoic acid; PFNA, perfluorononanoic acid; PFUdA, perfluorodecanoic acid; PFUdA, perfluoroundecanoic acid; PFHxS, perfluorohexane sulfonate; PFOS, perfluorooctane sulfonate. Models further adjusted for footnotes a-d.

a Total lipids (g/L).

b Total lipids (g/L), father's education level (<12 years, 12 years, >12 years) and passive tobacco smoking (yes/no).

c Total lipids (g/L), father's education level (<12 years, 12 years, >12 years).

d For father's education level (<12 years, 12 years, >12 years) and passive tobacco smoking (yes/no).

# *p* < 0.1

* *p* < 0.05

** *p* < 0.01.

**Table 6:** Associations between POP serum concentrations and TSH, fT3, and fT4 serum concentrations at age 12 years, among girls (*n* = 227)

	TSH (mlU log-transformed)		fT3 (pg/mL log-transformed)		fT4 (ng/dL log-transformed)	
Exposure (µg/L log-transformed)	ß	95% Cl	ß	95% Cl	ß	95% Cl
PCB118	−0.006	(−0.115, 0.103)^a^	−0.017	(−0.046, 0.011)^c^	0.000	(−0.026, 0.027)^c^
PCB138	−0.026	(−0.128, 0.075)^a^	−0.026	(−0.055, 0.003)^c^. ^#^	0.006	(−0.021, 0.033)^c^
PCB153	−0.013	(−0.106, 0.080)^a^	−0.021	(−0.048, 0.007)^c^	0.003	(−0.023, 0.029)^c^
PCB180	−0.003	(−0.078, 0.072)^a^	−0.019	(−0.042, 0.004)^c^	−0.001	(−0.022, 0.021)^c^
HCB	−0.149	(−0.359, 0.062)^a^	−0.054	(−0.111, 0.003)^c^. ^#^	0.033	(−0.020, 0.086)^c^
p,p'-DDE			−0.026	(−0.057, 0.004)^c^. ^#^	0.014	(−0.014, 0.042)^c^
p,p'-DDE (T2 vs. T1)	−0.047	(−0.201, 0.106)^a^				
p,p'-DDE (T3 vs. T1)	−0.086	(−0.238, 0.066)^a^				
Beta-HCH	0.085	(−0.022, 0.193)^a^	−0.012	(−0.042, 0.018)^c^		
Beta-HCH (T2 vs. T1)					−0.012	(−0.049, 0.025)^c^
Beta-HCH (T3 vs. T1)					0.015	(−0.025, 0.056)^c^
PFOA	−0.098	(−0.310, 0.115)^b^			0.009	(−0.042, 0.060)^c^
PFOA (T2 vs. T1)			−0.063	(−0.100, −0.026)^d^, **		
PFOA (T3 vs. T1)			−0.042	(−0.082, −0.002)^d^, *		
PFNA	−0.055	(−0.203, 0.093)	−0.012	(−0.050, 0.025)^d^	0.006	(−0.029, 0.040)^d^
PFUdA	0.001	(−0.139, 0.141)	0.005	(−0.031, 0.040)^d^	0.006	(−0.029, 0.038)^d^
PFDA	−0.026	(−0.205, 0.153)	−0.024	(−0.068, 0.021)^d^	0.007	(−0.034, 0.048)^d^
PFHxS	−0.149	(−0.294, −0.004)*	−0.019	(−0.056, 0.018)^d^	0.020	(−0.014, 0.054)^d^
PFOS	−0.130	(−0.285, 0.026)	0.000	(−0.040, 0.039)^d^		
PFOS (T2 vs. T1)					−0.003	(−0.038, 0.032)^d^
PFOS (T3 vs. T1)					0.008	(−0.029, 0.045)^d^

All models adjusted for parental history of thyroid disease, season and hour of blood drawing. HCB, hexachlorbenzene; p,p'-DDE, dichlorodiphenyldichloroethylene; beta-HCH, beta hexachlorocyclohexane; PFOA, perfluorooctanoic acid; PFNA, perfluorononanoic acid; PFDA, perfluorodecanoic acid; PFUdA, perfluoroundecanoic acid; PFHxS, perfluorohexane sulfonate; PFOS, perfluorooctane sulfonate. Models further adjusted for a-d.

a total lipids (g/L), father's education level (<12 years, 12 years, >12 years) and passive tobacco smoking (yes/no).

b For father's education level (<12 years, 12 years, >12 years) and passive tobacco smoking (yes/no).

c Total lipids (g/L), breastfeeding (none, ≤3 months, ≥4 months).

d Breastfeeding (none, ≤3 months, >3 months).

# *p* < 0.1

* *p* < 0.05

** *p* < 0.01.

**Table 7:** Associations between POP serum concentrations and TSH, serum concentrations at age 12 years according to pubertal status among boys (*n* = 223)

	TSH (mlU log-transformed)						
	Tanner 1-2 for genitalia (*n* = 74)		Tanner 3 for genitalia (*n* = 87)		Tanner 4-5 for genitalia (*n* = 62)		
Exposure (µg/L log-transformed)	ß	95% Cl	ß	95% Cl	ß	95% Cl	*p* int
PCB118	0.130	(−0.120, 0.381)	−0.142	(−0.367, 0.082)	−0.268	(−0.552, 0.017)^#^	^##^
PCB138	0.065	(−0.157, 0.286)	−0.205	(−0.423, 0.013)^#^	−0.214	(−0.469, 0.040)^#^	^#^
PCB153	0.045	(−0.138, 0.229)	−0.178	(−0.373, 0.017)^#^	−0.114	(−0.344, 0.116)	^##^
PCB180	0.050	(−0.098, 0.197)	−0.125	(−0.277, 0.027)	−0.041	(−0.218, 0.136)	^##^
HCB	0.054	(−0.381, 0.489)	−0.018	(−0.451, 0.415)	−0.560	(−1.101, −0.018)*	
p,p'-DDE p,p'-DDE (T2 vs. T1)	0.100	(−0.178, 0.378)	−0.197	(−0.432, 0.038)^#^	−0.012	(−0.382, 0.358)	
p,p'-DDE (T3 vs. T1)	−0.025	(−0.349, 0.298)	−0.095	(−0.349, 0.159)	−0.086	(−0.476, 0.304)	
Beta-HCH	0.037	(−0.147, 0.221)	−0.174	(−0.375, 0.027)^#^	−0.171	(−0.391, 0.050)	
PFOA	0.193	(−0.163, 0.550)	−0.092	(−0.374, 0.190)	−0.158	(−0.562, 0.246)	
PFNA	−0.010	(−0.399, 0.378)	−0.083	(−0.393, 0.227)	0.062	(−0.208, 0.333)	
PFUdA	−0.048	(−0.254, 0.157)	0.031	(−0.167, 0.230)	−0.127	(−0.376, 0.122)	
PFDA	−0.208	(−0.528, 0.113)	−0.025	(−0.249, 0.200)	−0.146	(−0.393, 0.101)	
PFHxS	0.233	(−0.051, 0.517)	0.037	(−0.197, 0.271)	−0.090	(−0.403, 0.223)	
PFOS	0.152	(−0.074, 0.378)	0.021	(−0.194, 0.236)	−0.249	(−0.482, −0.016)*	_*_

All models adjusted for parental history of thyroid disease, season and hour of blood drawing; and further adjusted for total lipids for PCBs, HCB, p,p'-DDE, and beta-HCH. *p* int: *p* value for interaction term between exposure and Tanner stage. HCB, hexachlorbenzene; p,p'-DDE, dichlorodiphenyldichloroethylene; beta-HCH, beta-hexachlorocyclohexane; PFOA, perfluorooctanoic acid; PFNA, perfluorononanoic acid; PFDA, perfluorodecanoic acid; PFUdA, perfluoroundecanoic acid; PFHxS, perfluorohexane sulfonate; PFOS, perfluorooctane sulfonate.

## *p* < 0.2

# *p* < 0.1

* *p* < 0.05.

**Table 8:** Associations between POP serum concentrations and fT3 and fT4 serum concentrations at age 12 years according to pubertal status among boys (*n* = 223)

	fT3 (pg/mF log-transformed) Tanner 1-2 for genitalia (*n****=*** 74)		Tanner 3 for genitalia (*n =* 87)		Tanner 4-5 for genitalia (*n* *=* 62)			fT4 (ng/dF log-transformed) Tanner 1-2 for genitalia (*n* ***=*** 74)		Tanner 3 for genitalia (*n* = 87)		Tanner 4-5 for genitalia (*n =* 62)		
Exposure (µg/L log-transformed)	p	95% Cl	p 95% Cl	P	95% Cl	p int	p	95% Cl	p	95% Cl	p	95% Cl p int
PCB118							−0.026	(−0.091, 0.040)	0.018	(−0.037, 0.072)	0.013	(−0.065, 0.092)
PCB118 (T2 vs. T1)	−0.010	(−0.082, 0.061)	−0.009 (0.057,0.039)	−0.052	(−0.109, 0.005)*	^##^						
PCB118 (T3 vs. T1)	−0.071	(−0.142, −0.001)*	−0.023 (−0.073,0.027)	0.017	(−0.037, 0.071)							
PCB138	−0.047	(−0.102, 0.008)*	0.001 (−0.044, 0.047)	−0.020	(−0.061, 0.021)	^##^	−0.021	(−0.079, 0.036)	0.003	(−0.051, 0.057)	0.025	(−0.044, 0.095)
PCB153							−0.011	(−0.059, 0.037)	0.005	(−0.043, 0.053)	0.017	(−0.045, 0.078)
PCB153 (T2 vs. T1)	−0.027	(−0.110,0.055)	−0.023 (−0.080,0.034)	−0.024	(−0.079, 0.032)							
PCB153 (T3 vs. T1)	−0.037	(−0.110,0.035)	−0.023 (−0.075,0.029)	−0.021	(−0.073,0.031)							
PCB180												
PCB180 (T2 vs. T1)	−0.056	(−0.134,0.023)	−0.029 (−0.085,0.027)	−0.013	(−0.068, 0.042)		−0.074	(−0.153,0.006)*	−0.065	(−0.129,0.000)^#^	0.042	(−0.052,0.137)
PCB180 (T3 vs. T1)	−0.044	(−0.116,0.028)	−0.032 (−0.086,0.023)	−0.022	(−0.077, 0.033)		−0.008	(−0.081,0.064)	−0.018	(−0.082, 0.045)	−0.003	(−0.098, 0.092)
HCB	−0.099	(−0.207, 0.009)^#^	−0.084 (−0.170,0.003)^#^	−0.020	(−0.108, 0.069)		−0.012	(−0.125,0.101)	−0.018	(−0.123, 0.086)	0.058	(−0.092, 0.209)
p,p'-DDE							−0.008	(−0.063, 0.047)	−0.013	(−0.065, 0.040)	0.062	(−0.002,0.127) ^#^
p,p'-DDE (T2 vs. T1)	−0.005	(−0.072, 0.063)	−0.001 (−0.050,0.047)	−0.028	(−0.085, 0.028)	^##^						
p,p'-DDE (T3 vs. T1)	−0.090	(−0.169,–0.011)*	−0.026 (−0.078,0.026)	−0.001	(−0.061,0.058)							
Beta-HCH	−0.023	(−0.069, 0.022)	−0.030 (−0.071,0.012)	0.004	(−0.031,0.040)							
Beta-HCH (T2 vs. T1)							−0.021	(−0.095, 0.053)	0.000	(−0.058, 0.057)	0.013	(−0.075,0.101)
Beta-HCH (T3 vs. T1)							0.000	(−0.079, 0.078)	0.026	(−0.038, 0.089)	0.029	(−0.068,0.126)
PFOA	0.089	(0.001,0.177)*	−0.009 (−0.067,0.049)	−0.035	(−0.099, 0.029)	#						
PFOA (T2 vs. T1)							−0.019	(−0.100,0.063)	0.009	(−0.049, 0.066)	0.012	(−0.088,0.113)
PFOA (T3 vs. T1)							0.034	(−0.040,0.108)	−0.011	(−0.074, 0.051)	−0.032	(−0.140,0.075)
PFNA	0.042	(−0.058,0.142)	−0.005 (−0.069,0.060)	−0.006	(−0.053, 0.040)		0.058	(−0.050,0.165)	0.026	(−0.053,0.105)	−0.021	(−0.106,0.065)
PFUdA							0.026	(−0.025, 0.077)	−0.019	(−0.067, 0.030)	−0.019	(−0.089,0.051)
PFUdA CT2 vs. T1)	0.000	(−0.071,0.071)	−0.010 (−0.059,0.040)	0.029	(−0.024, 0.082)							
PFUdA (T3 vs. T1)	0.012	(−0.078,0.101)	0.008 (−0.055,0.071)	−0.020	(−0.086, 0.046)							
PFDA	0.029	(−0.055,0.113)	−0.040 (−0.083,0.003)^#^	−0.014	(−0.053, 0.026)		0.000	(−0.089, 0.088)	0.023	(−0.033, 0.080)	−0.023	(−0.093, 0.048)
PFHxS	0.021	(−0.051,0.094)	−0.004 (−0.052,0.044)	0.004	(−0.046, 0.053)							
PFHxS (T2 vs. T1)							0.048	(−0.038,0.135)	0.005	(−0.057, 0.068)	0.019	(−0.086,0.123)
PFHxS (T3 vs. T1)							0.043	(−0.040,0.126)	−0.026	(−0.086, 0.034)	0.003	(−0.096,0.102)
PFOS	0.009	(−0.049, 0.066)	−0.031 (−0.074,0.013)	−0.018	(−0.056, 0.020)		−0.018	(−0.077,0.041)	0.001	(−0.051,0.054)	−0.017	(−0.086,0.051)

All models adjusted for parental history of thyroid disease, season and hour of blood drawing; and further adjusted for total lipids for PCBs, HCB, p,p'-DDE, and beta-HCH. p int: *p* value for interaction term between exposure and Tanner stage. HCB, hexachlorbenzene; p,p'-DDE, dichlorodiphenyldichloroethylene; beta-HCH, beta-hexachlorocyclohexane; PFOA, perfluorooctanoic acid; PFNA, perfluorononanoic acid; PFDA, perfluorodecanoic acid; PFUdA, perfluoroundecanoic acid; PFHxS, perfluorohexane sulfonate; PFOS, perfluorooctane sulfonate.

## p < 0.2

# p < 0.1

* *p <* 0.05.
