# Supplementary material for: Persistent Organic Pollutant Exposure and Thyroid Function among 12-Year-Old Children
Source: Neuroendocrinology. 2022 Dec 9;113(12):1232–47. doi: 10.1159/000528631 (PMC10906475; doi:10.1159/000528631)
Supplement: Supplementary file 1 — Supplementary data [file nen-0113-1232-s01.docx]

***Research Article***

***Persistent organic pollutant exposure***

***and thyroid function among 12-year-old children***

Hélène Tillaut^a^, Christine Monfort^a^, Frank Giton^b^, Charline Warembourg^a^, Florence Rouget^c^, Sylvaine Cordier^a^, Fabrice Lainé^d^, Eric Gaudreau^e^, Ronan Garlantézec^c^, Dave Saint-Amour^f^, Cécile Chevrier^a^

^a^ Univ Rennes, Inserm, EHESP, Irset (Institut de recherche en santé, environnement et travail) - UMR_S 1085, F-35000 Rennes, France

^b^ AP-HP, Pôle Biologie-Pathologie Henri Mondor, Créteil, France ; Inserm IMRB, Faculté de Santé, Créteil, France

^c^ Univ Rennes, CHU Rennes, Inserm, EHESP, Irset (Institut de recherche en santé, environnement et travail) - UMR_S 1085, F-35000 Rennes, France

^d^ CHU Rennes, INSERM CIC1414, F-35000, Rennes, France

^e^ Centre de Toxicologie du Québec (CTQ), Institut national de santé publique du Québec (INSPQ), Québec, Canada

^f^ Département de Psychologie, Université du Québec à Montréal, Montréal, Canada ; Centre de Recherche du Centre Hospitalier Universitaire Sainte-Justine, Montréal, Canada

**Supplemental material**

**
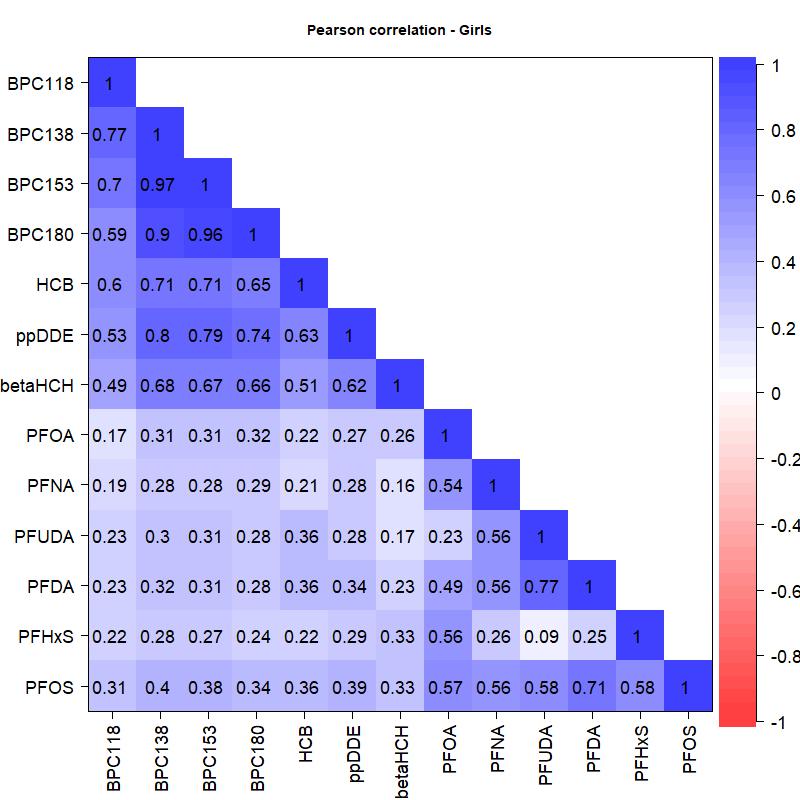

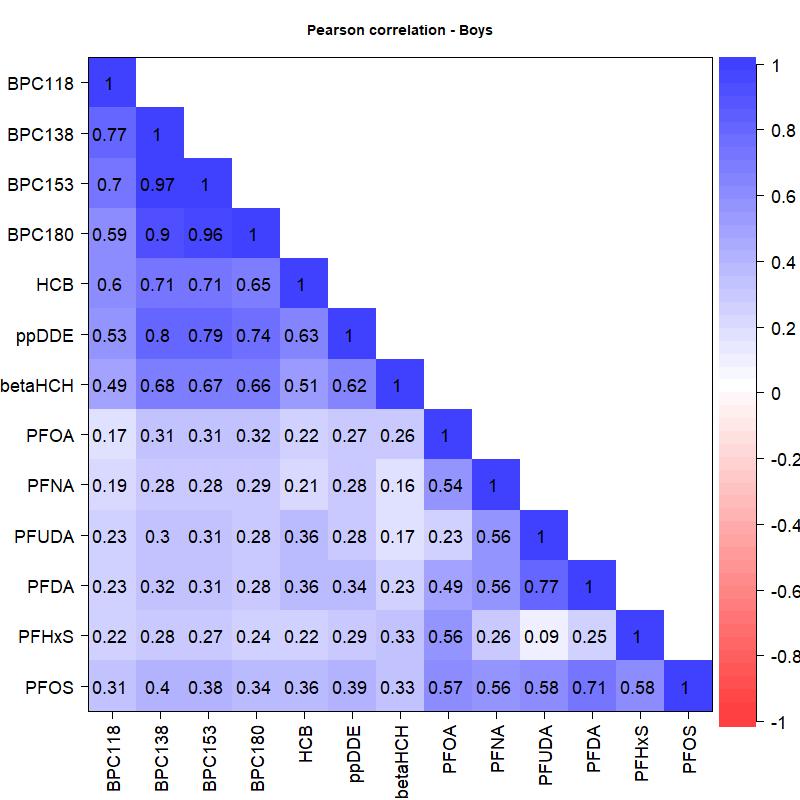
Figure S1 - Pearson correlations of POP concentrations at age 12**

**Figure S2 – Shape of the relationship between thyroid hormones and exposures – restricted cubic splines**


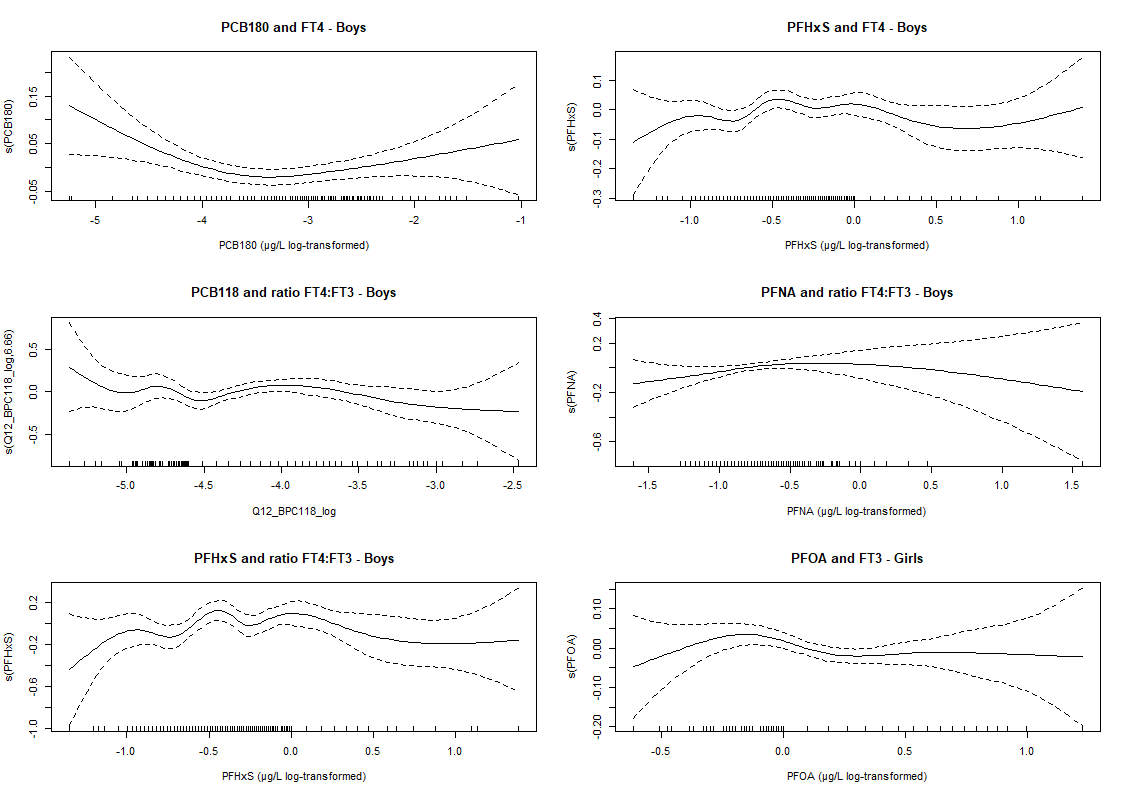


**Table S1. Associations between POP serum concentrations and TSH, free T3 and free T4 serum concentrations at age 12. Minimal set of confounders.**

|  | **TSH (log-transformed)** | | | | |  |  |  | **free T3 (log-transformed)** | | | | | |  |  | **free T4 (log-transformed)** | | | | | |  | |  | |  |
| --- | --- | --- | --- | --- | --- | --- | --- | --- | --- | --- | --- | --- | --- | --- | --- | --- | --- | --- | --- | --- | --- | --- | --- | --- | --- | --- | --- |
|  | **Boys** |  |  |  | **Girls** |  |  |  | **Boys** |  |  |  | **Girls** |  |  |  | **Boys** |  |  |  | **Girls** |  | |  | |  | |
| **Exposure (log-transformed)** | **β** | **95%CI** | **p** |  | **β** | **95%CI** | **p** |  | **β** | **95%CI** | **p** |  | **β** | **95%CI** | **p** |  | **β** | **95%CI** | **p** |  | **β** | **95%CI** | | **p** | |  | |
| PCB118 | -0.068 | (-0.192,0.057) |  | ^a^ | -0.041 | (-0.151,0.069) |  | ^a^ |  |  |  |  | -0.005 | (-0.033,0.023) |  | ^a^ | -0.015 | (-0.048,0.019) |  | ^a^ | 0.008 | (-0.017,0.034) | |  | | ^a^ | |
| PCB118 (T2 vs. T1) |  |  |  |  |  |  |  |  | -0.026 | (-0.059,0.007) |  | ^a^ |  |  |  |  |  |  |  |  |  |  | |  | |  | |
| PCB118 (T3 vs. T1) |  |  |  |  |  |  |  |  | -0.025 | (-0.057,0.006) |  | ^a^ |  |  |  |  |  |  |  |  |  |  | |  | |  | |
| PCB138 | -0.072 | (-0.188,0.045) |  | ^a^ |  |  |  |  | -0.011 | (-0.037,0.015) |  | ^a^ | 0.000 | (-0.026,0.026) |  | ^a^ | -0.008 | (-0.039,0.023) |  | ^a^ | 0.018 | (-0.005,0.042) | |  | | ^a^ | |
| PCB138 (T2 vs. T1) |  |  |  |  | -0.163 | (-0.315,-0.012) | * | ^c^ |  |  |  |  |  |  |  |  |  |  |  |  |  |  | |  | |  | |
| PCB138 (T3 vs. T1) |  |  |  |  | -0.122 | (-0.275,0.030) |  | ^c^ |  |  |  |  |  |  |  |  |  |  |  |  |  |  | |  | |  | |
| PCB153 | -0.047 | (-0.149,0.055) |  | ^a^ | -0.052 | (-0.143,0.039) |  | ^a^ |  |  |  |  | 0.006 | (-0.017,0.030) |  | ^a^ | -0.004 | (-0.031,0.023) |  | ^a^ | 0.016 | (-0.005,0.037) | |  | | ^a^ | |
| PCB153 (T2 vs. T1) |  |  |  |  |  |  |  |  | -0.023 | (-0.055,0.009) |  | ^a^ |  |  |  |  |  |  |  |  |  |  | |  | |  | |
| PCB153 (T3 vs. T1) |  |  |  |  |  |  |  |  | -0.009 | (-0.041,0.024) |  | ^a^ |  |  |  |  |  |  |  |  |  |  | |  | |  | |
| PCB180 | -0.022 | (-0.103,0.059) |  | ^a^ | -0.037 | (-0.110,0.037) |  | ^a^ |  |  |  |  | 0.006 | (-0.013,0.025) |  | ^a^ |  |  |  |  | 0.012 | (-0.005,0.029) | |  | | ^a^ | |
| PCB180 (T2 vs. T1) |  |  |  |  |  |  |  |  | -0.027 | (-0.059,0.005) | . | ^a^ |  |  |  |  | -0.041 | (-0.080,-0.002) | * | ^a^ |  |  | |  | |  | |
| PCB180 (T3 vs. T1) |  |  |  |  |  |  |  |  | -0.019 | (-0.052,0.014) |  | ^a^ |  |  |  |  | -0.004 | (-0.044,0.036) |  |  |  |  | |  | |  | |
| Hexchlorobenzene | -0.149 | (-0.378,0.080) |  | ^a^ | -0.207 | (-0.416,0.003) | . | ^a^ | -0.063 | (-0.113,-0.014) | * | ^a^ | -0.013 | (-0.067,0.040) |  | ^a^ | -0.031 | (-0.092,0.030) |  |  | 0.050 | (0.001,0.099) | | * | | ^a^ | |
| ppDDE |  |  |  |  |  |  |  |  |  |  |  |  | -0.002 | (-0.029,0.026) |  | ^a^ | 0.003 | (-0.026,0.033) |  |  | 0.025 | (0.000,0.050) | | . | | ^a^ | |
| ppDDE (T2 vs. T1) | -0.015 | (-0.159,0.130) |  | ^a^ | -0.087 | (-0.243,0.069) |  | ^a^ | 0.007 | (-0.025,0.038) |  | ^a^ |  |  |  |  |  |  |  |  |  |  | |  | |  | |
| ppDDE (T3 vs. T1) | -0.068 | (-0.225,0.090) |  | ^a^ | -0.144 | (-0.296,0.008) | . | ^a^ | -0.015 | (-0.050,0.019) |  | ^a^ |  |  |  |  |  |  |  |  |  |  | |  | |  | |
| betaHCH | -0.087 | (-0.189,0.016) | . | ^a^ | 0.066 | (-0.042,0.175) |  | ^a^ | -0.010 | (-0.032,0.013) |  | ^a^ | 0.007 | (-0.021,0.034) |  | ^a^ |  |  |  |  |  |  | |  | |  | |
| betaHCH (T2 vs. T1) |  |  |  |  |  |  |  |  |  |  |  |  |  |  |  |  | -0.007 | (-0.044,0.031) |  | ^a^ | -0.002 | (-0.037,0.034) | |  | | ^a^ | |
| betaHCH (T3 vs. T1) |  |  |  |  |  |  |  |  |  |  |  |  |  |  |  |  | 0.023 | (-0.018,0.064) |  | ^a^ | 0.030 | (-0.009,0.068) | |  | | ^a^ | |
| PFOA | -0.002 | (-0.178,0.174) |  |  | -0.110 | (-0.328,0.108) |  |  | -0.011 | (-0.050,0.028) |  |  |  |  |  |  |  |  |  |  | 0.019 | (-0.031,0.070) | |  | |  | |
| PFOA (T2 vs. T1) |  |  |  |  |  |  |  |  |  |  |  |  | -0.063 | (-0.101,-0.025) | ** |  | 0.012 | (-0.026,0.050) |  |  |  |  | |  | |  | |
| PFOA (T3 vs. T1) |  |  |  |  |  |  |  |  |  |  |  |  | -0.035 | (-0.076,0.005) | . |  | 0.021 | (-0.019,0.060) |  |  |  |  | |  | |  | |
| PFNA | 0.072 | (-0.091,0.234) |  |  | -0.055 | (-0.203,0.093) |  |  | -0.006 | (-0.043,0.031) |  |  | -0.005 | (-0.042,0.033) |  |  | 0.022 | (-0.024,0.068) |  |  | 0.011 | (-0.023,0.045) | |  | |  | |
| PFUDA | -0.041 | (-0.153,0.072) |  |  | 0.001 | (-0.139,0.141) |  |  |  |  |  |  | 0.011 | (-0.024,0.047) |  |  | 0.000 | (-0.029,0.030) |  |  | 0.010 | (-0.022,0.042) | |  | |  | |
| PFUDA (T2 vs. T1) |  |  |  |  |  |  |  |  | 0.010 | (-0.022,0.041) |  |  |  |  |  |  |  |  |  |  |  |  | |  | |  | |
| PFUDA (T3 vs. T1) |  |  |  |  |  |  |  |  | -0.004 | (-0.044,0.035) |  |  |  |  |  |  |  |  |  |  |  |  | |  | |  | |
| PFDA | -0.108 | (-0.242,0.027) |  |  | -0.026 | (-0.205,0.153) |  |  | -0.031 | (-0.060,-0.001) | * |  | -0.018 | (-0.064,0.027) |  |  | 0.012 | (-0.025,0.048) |  |  | 0.010 | (-0.031,0.052) | |  | |  | |
| PFHxS | 0.056 | (-0.087,0.198) |  |  | -0.149 | (-0.294,-0.004) | * |  | -0.009 | (-0.040,0.022) |  |  | -0.013 | (-0.050,0.025) |  |  |  |  |  |  | 0.024 | (-0.010,0.058) | |  | |  | |
| PFHxS (T2 vs. T1) |  |  |  |  |  |  |  |  |  |  |  |  |  |  |  |  | 0.039 | (0.000,0.078) | * |  |  |  | |  | |  | |
| PFHxS (T3 vs. T1) |  |  |  |  |  |  |  |  |  |  |  |  |  |  |  |  | 0.022 | (-0.017,0.061) |  |  |  |  | |  | |  | |
| PFOS | -0.047 | (-0.167,0.072) |  |  | -0.130 | (-0.285,0.026) |  |  | -0.015 | (-0.041,0.011) |  |  | 0.004 | (-0.036,0.044) |  |  | 0.002 | (-0.030,0.033) |  |  |  |  | |  | |  | |
| PFOS (T2 vs. T1) |  |  |  |  |  |  |  |  |  |  |  |  |  |  |  |  |  |  |  |  | 0.000 | (-0.035,0.035) | |  | |  | |
| PFOS (T3 vs. T1) |  |  |  |  |  |  |  |  |  |  |  |  |  |  |  |  |  |  |  |  | 0.013 | (-0.024,0.049) | |  | |  | |

*All models adjusted for parental history of thyroid disease, season and hour of blood drawing; ^a^ Models further adjusted for total lipids (g/L); p,p'-DDE: dichlorodiphenyldichloroethylene; beta-HCH: beta hexachlorocyclohexane; PFOA: perfluorooctanoic acid; PFNA: perfluorononanoic acid; PFDA: perfluorodecanoic acid; PFUdA: perfluoroundecanoic acid; PFHxS: perfluorohexane sulfonate; PFOS: perfluorooctane sulfonate*

**Table S2. Associations between POP serum concentrations and free T4 : free T3 ratio at age 12.**

|  | **Ratio FT4/FT3 (molar weight)** | | | | | | | |
| --- | --- | --- | --- | --- | --- | --- | --- | --- |
|  | **Boys** | |  |  | **Girls** | |  |  |
| **Exposure (µg/L log-transformed)** | **β** | **95%CI** | **p** |  | **β** | **95%CI** | **p** |  |
| PCB118 |  |  |  | ^a^ | 0.042 | (-0.037,0.122) |  | ^d^ |
| PCB118 (T2 vs. T1) | 0.122 | (0.005,0.239) | * |  |  |  |  |  |
| PCB118 (T3 vs. T1) | 0.042 | (-0.071,0.154) |  |  |  |  |  |  |
| PCB138 | 0.014 | (-0.078,0.105) |  | ^a^ | 0.072 | (-0.009,0.153) | . | ^d^ |
| PCB153 | 0.011 | (-0.069,0.092) |  | ^a^ | 0.051 | (-0.026,0.129) |  | ^d^ |
| PCB180 | 0.016 | (-0.048,0.080) |  | ^a^ | 0.038 | (-0.027,0.103) |  | ^d^ |
| Hexachlorobenzene | 0.085 | (-0.095,0.265) |  |  | 0.202 | (0.045,0.359) | * | ^d^ |
| p,p'-DDE | 0.025 | (-0.062,0.112) |  |  | 0.091 | (0.007,0.175) | * | ^d^ |
| beta-HCH | 0.033 | (-0.047,0.113) |  | ^a^ | 0.014 | (-0.070,0.097) |  | ^d^ |
| PFOA |  |  |  |  | 0.153 | (-0.002,0.307) | . |  |
| PFOA (T2 vs. T1) | 0.090 | (-0.020,0.199) |  |  |  |  |  | ^f^ |
| PFOA (T3 vs. T1) | 0.099 | (-0.014,0.212) | . |  |  |  |  |  |
| PFNA |  |  |  |  | 0.033 | (-0.072,0.138) |  | ^f^ |
| PFNA (T2 vs. T1) | 0.159 | (0.043,0.275) | ** |  |  |  |  |  |
| PFNA (T3 vs. T1) | 0.115 | (-0.002,0.232) | . |  |  |  |  |  |
| PFUDA |  |  |  |  | 0.003 | (-0.096,0.101) |  | ^f^ |
| PFUDA (T2 vs. T1) | 0.036 | (-0.074,0.146) |  |  |  |  |  |  |
| PFUDA (T3 vs. T1) | 0.050 | (-0.087,0.188) |  |  |  |  |  |  |
| PFDA | 0.116 | (0.013,0.220) | * |  | 0.067 | (-0.058,0.193) |  | ^f^ |
| PFHxS |  |  |  |  | 0.089 | (-0.014,0.191) | . | ^f^ |
| PFHxS (T2 vs. T1) | 0.105 | (-0.007,0.218) | . |  |  |  |  |  |
| PFHxS (T3 vs. T1) | 0.115 | (0.001,0.228) | * |  |  |  |  |  |
| PFOS | 0.052 | (-0.042,0.146) |  |  |  |  |  |  |
| PFOS (T2 vs. T1) |  |  |  |  | 0.011 | (-0.001,0.024) | . | ^f^ |
| PFOS (T3 vs. T1) |  |  |  |  | 0.004 | (-0.009,0.017) |  | ^f^ |

*All models adjusted for parental history of thyroid disease, season and hour of blood drawing; . p<0.1; * p<0.05; ** p<0.01. Molar weight Triiodothyronine (FT3): 650.98 g·mol-1; Molar weight Thyroxine (FT4): 776.87 g·mol-1. ^a^ Models further adjusted for total lipids (g/L); ^b^ Models further adjusted for total lipids (g/L), father's education level (< 12 years, 12 years, > 12 years); ^c^ Models further adjusted for total lipids (g/L), father's education level (< 12 years, 12 years, > 12 years) and passive tobacco smoking (yes/no); ^d^ Models further adjusted for total lipids (g/L), breastfeeding (none, ≤ 3 months, ≥ 4 months); ^e^ Models further adjusted for father's education level (< 12 years, 12 years, > 12 years) and passive tobacco smoking (yes/no); ^f^ Models further adjusted for breastfeeding (none, ≤ 3 months, ≥ 4 months); p,p'-DDE: dichlorodiphenyldichloroethylene; beta-HCH: beta hexachlorocyclohexane; PFOA: perfluorooctanoic acid; PFNA: perfluorononanoic acid; PFDA: perfluorodecanoic acid; PFUdA: perfluoroundecanoic acid; PFHxS: perfluorohexane sulfonate; PFOS: perfluorooctane sulfonate*

**Table S3 - POP cord serum concentrations**

| **POP** | **LOD (µg/L)** | **N** | **% ND** | **Q10 (µg/L)** | **Q25 (µg/L)** | **Q50 (µg/L)** | **Q75 (µg/L)** | **Q90 (µg/L)** |
| --- | --- | --- | --- | --- | --- | --- | --- | --- |
| PCB118 | 0.01 | 449 | 1.11 | 0.02 | 0.02 | 0.03 | 0.04 | 0.06 |
| PCB138 | 0.01 | 436 | 0.00 | 0.04 | 0.05 | 0.07 | 0.10 | 0.13 |
| PCB153 | 0.01 | 449 | 0.00 | 0.06 | 0.09 | 0.12 | 0.17 | 0.24 |
| PCB180 | 0.01 | 449 | 0.00 | 0.04 | 0.06 | 0.08 | 0.11 | 0.16 |
| Hexachlorobenzene | 0.02 | 449 | 8.91 | 0.02 | 0.03 | 0.04 | 0.06 | 0.07 |
| p,p'-DDE | 0.02 | 449 | 6.46 | 0.07 | 0.12 | 0.19 | 0.33 | 0.49 |
| Beta-HCH | 0.01 | 449 | 1.34 | 0.03 | 0.04 | 0.05 | 0.08 | 0.12 |
| PFOA | 0.07 | 399 | 0.00 | 1.30 | 1.70 | 2.30 | 2.80 | 3.70 |
| PFNA | 0.09 | 390 | 0.26 | 0.20 | 0.28 | 0.35 | 0.47 | 0.58 |
| PFUDA | 0.05 | 399 | 24.56 | <0.05 | 0.05 | 0.07 | 0.09 | 0.1 |
| PFDA | 0.06 | 393 | 8.40 | 0.06 | 0.08 | 0.1 | 0.1 | 0.2 |
| PFHxS | 0.06 | 399 | 0.00 | 0.31 | 0.42 | 0.55 | 0.70 | 0.95 |
| PFOS | 0.43 | 399 | 0.00 | 2.80 | 3.50 | 4.50 | 5.90 | 7.60 |

*LOD: limit of detection; ND: not detected*

*p,p'-DDE: dichlorodiphenyldichloroethylene; beta-HCH: beta hexachlorocyclohexane; PFOA: perfluorooctanoic acid; PFNA: perfluorononanoic acid; PFDA: perfluorodecanoic acid; PFUdA: perfluoroundecanoic acid; PFHxS: perfluorohexane sulfonate; PFOS: perfluorooctane sulfonate*

**Table S4. Associations between POP serum concentrations and TSH, free T3 and free T4 serum concentrations at age 12. Complete set of confounders. Complementary adjustment for cord-serum POP exposure – Boys**

|  | **TSH (mIU log-transformed)** | | | | | |  |  | **free T3 (pg/mL log-transformed)** | | | | | |  |  | **free T4 (ng/dL log-transformed)** | | | | | |  |  |
| --- | --- | --- | --- | --- | --- | --- | --- | --- | --- | --- | --- | --- | --- | --- | --- | --- | --- | --- | --- | --- | --- | --- | --- | --- |
|  | **Boys^ⱡ^** |  |  |  | **Boys^ⱡⱡ^** |  |  |  | **Boys^ⱡ^** |  |  |  | **Boys^ⱡⱡ^** |  |  |  | **Boys^ⱡ^** |  |  |  | **Boys^ⱡⱡ^** |  |  |  |
| **Exposure (µg/L log-transformed)** | **β** | **95%CI** | **p** |  | **β** | **95%CI** | **p** |  | **β** | **95%CI** | **p** |  | **β** | **95%CI** | **p** |  | **β** | **95%CI** | **p** |  | **β** | **95%CI** | **p** |  |
| PCB118 | -0.051 | (-0.181,0.080) |  | ^a^ | -0.088 | (-0.227,0.051) |  | ^a^ |  |  |  |  |  |  |  |  | -0.005 | (-0.039,0.030) |  | ^a^ | -0.005 | (-0.042,0.032) |  | ^a^ |
| PCB118 (T2 vs. T1) |  |  |  |  |  |  |  |  | -0.030 | (-0.066,0.005) | . | ^c^ | -0.033 | (-0.068,0.003) | . | ^c^ |  |  |  |  |  |  |  |  |
| PCB118 (T3 vs. T1) |  |  |  |  |  |  |  |  | -0.026 | (-0.060,0.008) |  | ^c^ | -0.033 | (-0.068,0.001) | . | ^c^ |  |  |  |  |  |  |  |  |
| PCB138 | -0.075 | (-0.203,0.053) |  | ^a^ | -0.085 | (-0.214,0.045) |  | ^a^ | -0.017 | (-0.045,0.012) |  | ^c^ | -0.018 | (-0.047,0.011) |  | ^c^ | 0.001 | (-0.032,0.035) |  | ^a^ | 0.001 | (-0.034,0.036) |  | ^a^ |
| PCB153 | -0.050 | (-0.160,0.060) |  | ^a^ | -0.072 | (-0.181,0.038) |  | ^a^ |  |  |  |  |  |  |  |  | -0.001 | (-0.030,0.028) |  | ^a^ | -0.001 | (-0.031,0.029) |  | ^a^ |
| PCB153 (T2 vs. T1) |  |  |  |  |  |  |  |  | -0.021 | (-0.055,0.013) |  | ^c^ | -0.018 | (-0.052,0.017) |  | ^c^ |  |  |  |  |  |  |  |  |
| PCB153 (T3 vs. T1) |  |  |  |  |  |  |  |  | -0.007 | (-0.041,0.028) |  | ^c^ | -0.014 | (-0.050,0.021) |  | ^c^ |  |  |  |  |  |  |  |  |
| PCB180 | -0.020 | (-0.108,0.067) |  | ^a^ | -0.042 | (-0.129,0.046) |  | ^a^ |  |  |  |  |  |  |  |  |  |  |  |  |  |  |  |  |
| PCB180 (T2 vs. T1) |  |  |  |  |  |  |  |  | -0.029 | (-0.063,0.005) | . | ^c^ | -0.029 | (-0.063,0.005) | . | ^c^ | -0.035 | (-0.076,0.007) | . | ^a^ | -0.037 | (-0.079,0.005) | . | ^a^ |
| PCB180 (T3 vs. T1) |  |  |  |  |  |  |  |  | -0.024 | (-0.060,0.012) |  | ^c^ | -0.026 | (-0.062,0.011) |  | ^c^ | -0.004 | (-0.047,0.038) |  |  | -0.002 | (-0.047,0.042) |  |  |
| Hexachlorobenzene | -0.179 | (-0.417,0.058) |  | ^a^ | -0.173 | (-0.411,0.066) |  | ^a^ | -0.078 | (-0.130,-0.027) | ** | ^b^ | -0.075 | (-0.127,-0.024) | ** | ^b^ | -0.018 | (-0.081,0.045) |  |  | -0.018 | (-0.082,0.045) |  |  |
| p,p'-DDE |  |  |  |  |  |  |  |  |  |  |  |  |  |  |  |  | 0.006 | (-0.027,0.038) |  |  | 0.006 | (-0.027,0.039) |  |  |
| p,p'-DDE (T2 vs. T1) | -0.020 | (-0.175,0.135) |  | ^a^ | -0.022 | (-0.177,0.134) |  | ^a^ | 0.019 | (-0.015,0.053) |  | ^b^ | 0.019 | (-0.015,0.053) |  | ^b^ |  |  |  |  |  |  |  |  |
| p,p'-DDE (T3 vs. T1) | -0.078 | (-0.245,0.090) |  | ^a^ | -0.099 | (-0.271,0.072) |  | ^a^ | -0.007 | (-0.043,0.030) |  | ^b^ | -0.010 | (-0.048,0.027) |  | ^b^ |  |  |  |  |  |  |  |  |
| beta-HCH | -0.137 | (-0.251,-0.022) | * | ^a^ | -0.153 | (-0.275,-0.031) | * | ^a^ | -0.017 | (-0.042,0.008) |  | ^b^ | -0.024 | (-0.050,0.003) | . | ^b^ |  |  |  |  |  |  |  |  |
| beta-HCH (T2 vs. T1) |  |  |  |  |  |  |  |  |  |  |  |  |  |  |  |  | 0.005 | (-0.036,0.045) |  | ^a^ | 0.002 | (-0.040,0.043) |  | ^a^ |
| beta-HCH (T3 vs. T1) |  |  |  |  |  |  |  |  |  |  |  |  |  |  |  |  | 0.030 | (-0.013,0.074) |  | ^a^ | 0.024 | (-0.022,0.070) |  | ^a^ |
| PFOA | 0.002 | (-0.195,0.199) |  |  | -0.004 | (-0.200,0.193) |  |  | -0.023 | (-0.065,0.018) |  | ^e^ | -0.026 | (-0.068,0.016) |  | ^e^ |  |  |  |  |  |  |  |  |
| PFOA (T2 vs. T1) |  |  |  |  |  |  |  |  |  |  |  |  |  |  |  |  | 0.005 | (-0.038,0.047) |  |  | 0.004 | (-0.039,0.047) |  |  |
| PFOA (T3 vs. T1) |  |  |  |  |  |  |  |  |  |  |  |  |  |  |  |  | 0.017 | (-0.027,0.060) |  |  | 0.017 | (-0.027,0.060) |  |  |
| PFNA | 0.094 | (-0.088,0.276) |  |  | 0.104 | (-0.078,0.286) |  |  | -0.025 | (-0.065,0.014) |  | ^e^ | -0.029 | (-0.069,0.011) |  | ^e^ | 0.013 | (-0.038,0.064) |  |  | 0.014 | (-0.037,0.066) |  |  |
| PFUDA | -0.042 | (-0.202,0.118) |  |  | -0.022 | (-0.187,0.143) |  |  |  |  |  |  |  |  |  |  | -0.001 | (-0.043,0.040) |  |  | 0.002 | (-0.041,0.044) |  |  |
| PFUDA (T2 vs. T1) |  |  |  |  |  |  |  |  | -0.009 | (-0.043,0.025) |  |  | -0.010 | (-0.044,0.025) |  |  |  |  |  |  |  |  |  |  |
| PFUDA (T3 vs. T1) |  |  |  |  |  |  |  |  | -0.030 | (-0.074,0.014) |  |  | -0.032 | (-0.076,0.013) |  |  |  |  |  |  |  |  |  |  |
| PFDA | -0.096 | (-0.252,0.060) |  |  | -0.062 | (-0.220,0.095) |  |  | -0.046 | (-0.079,-0.014) | ** | ^e^ | -0.053 | (-0.086,-0.020) | ** | ^e^ | -0.001 | (-0.043,0.042) |  |  | 0.000 | (-0.043,0.044) |  |  |
| PFHxS | 0.066 | (-0.096,0.228) |  |  | 0.042 | (-0.121,0.205) |  |  | -0.011 | (-0.046,0.024) |  | ^e^ | -0.010 | (-0.046,0.025) |  | ^e^ |  |  |  |  |  |  |  |  |
| PFHxS (T2 vs. T1) |  |  |  |  |  |  |  |  |  |  |  |  |  |  |  |  | 0.036 | (-0.007,0.080) |  |  | 0.038 | (-0.006,0.082) | . |  |
| PFHxS (T3 vs. T1) |  |  |  |  |  |  |  |  |  |  |  |  |  |  |  |  | 0.030 | (-0.014,0.073) |  |  | 0.032 | (-0.012,0.076) |  |  |
| PFOS | -0.045 | (-0.185,0.096) |  |  | -0.050 | (-0.194,0.094) |  |  | -0.021 | (-0.051,0.009) |  | ^e^ | -0.021 | (-0.051,0.010) |  | ^e^ | 0.017 | (-0.020,0.054) |  |  | 0.019 | (-0.019,0.057) |  |  |
| *All models adjusted for parental history of thyroid disease, season and hour of blood drawing* | | | | | | | | | | | | | | | | | | | | | | | | |
| *a Models further adjusted for total lipids (g/L), b Models further adjusted for total lipids (g/L), father's education level (< 12 years, 12 years, > 12 years), c Models further adjusted for total lipids (g/L), father's education level (< 12 years, 12 years, > 12 years) and passive tobacco smoking (yes/no), d Models further adjusted for total lipids (g/L), breastfeeding (none, ≤ 3 months, > 3 months), e Models further adjusted for father's education level (< 12 years, 12 years, > 12 years) and passive tobacco smoking (yes/no), f Models further adjusted for breastfeeding (none, ≤ 3 months, > 3 months), ⱡ Models adjusted for 12-year-old exposure, ⱡⱡ Models adjusted for 12-year-old POP serum concentration and POP cord-serum concentration, p,p'-DDE: dichlorodiphenyldichloroethylene; beta-HCH: beta hexachlorocyclohexane; PFOA: perfluorooctanoic acid; PFNA: perfluorononanoic acid; PFDA: perfluorodecanoic acid; PFUdA: perfluoroundecanoic acid; PFHxS: perfluorohexane sulfonate; PFOS: perfluorooctane sulfonate* | | | | | | | | | | | | | | | | | | | | | | | | |

**Table S5. Associations between POP serum concentrations and TSH, free T3 and free T4 serum concentrations at age 12. Complete set of confounders. Complementary adjustment for cord-serum POP exposure – Girls**

|  | | **TSH (mIU log-transformed)** | | | | |  | |  |  |  | **free T3 (pg/mL log-transformed)** | | | | |  |  |  | **free T4 (ng/dL log-transformed)** | | | | |  |  |  | |
| --- | --- | --- | --- | --- | --- | --- | --- | --- | --- | --- | --- | --- | --- | --- | --- | --- | --- | --- | --- | --- | --- | --- | --- | --- | --- | --- | --- | --- |
|  | | **Girls^ⱡ^** |  |  | |  | | **Girls^ⱡⱡ^** |  |  |  | **Girls^ⱡ^** |  |  |  | **Girls^ⱡⱡ^** |  |  |  | **Girls^ⱡ^** |  |  |  | **Girls^ⱡⱡ^** |  |  |  | |
| **Exposure (µg/L log-transformed)** | | **β** | **95%CI** | **p** | |  | | **β** | **95%CI** | **p** |  | **β** | **95%CI** | **p** |  | **β** | **95%CI** | **p** |  | **β** | **95%CI** | **p** |  | **β** | **95%CI** | **p** |  | |
| PCB118 | | 0.011 | (-0.103,0.126) |  | | ^c^ | | 0.023 | (-0.096,0.141) |  | ^c^ | -0.021 | (-0.051,0.009) |  | ^d^ | -0.021 | (-0.052,0.010) |  | ^d^ | -0.005 | (-0.032,0.022) |  | ^d^ | -0.007 | (-0.035,0.022) |  | ^d^ | |
| PCB138 | |  |  |  | |  | |  |  |  |  | -0.027 | (-0.059,0.004) | . | ^d^ | -0.033 | (-0.067,0.001) | . | ^d^ | -0.002 | (-0.030,0.027) |  | ^d^ | -0.010 | (-0.041,0.022) |  | ^d^ | |
| PCB138 (T2 vs. T1) | | -0.092 | (-0.258,0.074) | | ^c^ | | -0.077 | | (-0.246,0.092) |  | ^c^ |  |  |  |  |  |  |  |  |  |  |  |  |  |  |  |  | |
| PCB138 (T3 vs. T1) | | -0.051 | (-0.220,0.118) | | ^c^ | | -0.026 | | (-0.201,0.149) |  | ^c^ |  |  |  |  |  |  |  |  |  |  |  |  |  |  |  |  | |
| PCB153 | | 0.008 | (-0.091,0.107) |  | | ^c^ | | 0.035 | (-0.073,0.142) |  | ^c^ | -0.024 | (-0.053,0.005) |  | ^d^ | -0.029 | (-0.061,0.003) | . | ^d^ | -0.007 | (-0.033,0.020) |  | ^d^ | -0.013 | (-0.042,0.016) |  | ^d^ | |
| PCB180 | | 0.012 | (-0.068,0.093) |  | | ^c^ | | 0.038 | (-0.049,0.126) |  | ^c^ | -0.024 | (-0.048,0.001) | . | ^d^ | -0.027 | (-0.054,0.001) | . | ^d^ | -0.010 | (-0.033,0.012) |  | ^d^ | -0.012 | (-0.036,0.013) |  | ^d^ | |
| Hexachlorobenzene | | -0.117 | (-0.342,0.108) |  | | ^c^ | | -0.108 | (-0.338,0.121) |  | ^c^ | -0.054 | (-0.114,0.007) | . | ^d^ | -0.054 | (-0.114,0.007) | . | ^d^ | 0.025 | (-0.030,0.081) |  | ^d^ | 0.027 | (-0.029,0.083) |  | ^d^ | |
| p,p'-DDE | |  |  |  | |  | |  |  |  |  | -0.026 | (-0.059,0.006) |  | ^d^ | -0.027 | (-0.062,0.008) |  | ^d^ | 0.016 | (-0.013,0.046) |  | ^d^ | 0.012 | (-0.020,0.044) |  | ^d^ | |
| p,p'-DDE (T2 vs. T1) | | -0.088 | (-0.253,0.077) |  | | ^c^ | | -0.102 | (-0.271,0.067) |  | ^c^ |  |  |  |  |  |  |  |  |  |  |  |  |  |  |  |  | |
| p,p'-DDE (T3 vs. T1) | | -0.099 | (-0.262,0.063) |  | | ^c^ | | -0.119 | (-0.289,0.050) |  | ^c^ |  |  |  |  |  |  |  |  |  |  |  |  |  |  |  |  | |
| beta-HCH | | 0.114 | (-0.002,0.230) | . | | ^c^ | | 0.133 | (0.014,0.252) | * | ^c^ | -0.009 | (-0.041,0.023) |  | ^d^ | -0.014 | (-0.047,0.020) |  | ^d^ |  |  |  |  |  |  |  |  | |
| beta-HCH (T2 vs. T1) | |  |  |  | |  | |  |  |  |  |  |  |  |  |  |  |  |  | -0.008 | (-0.047,0.030) |  | ^d^ | -0.007 | (-0.047,0.032) |  | ^d^ | |
| beta-HCH (T3 vs. T1) | |  |  |  | |  | |  |  |  |  |  |  |  |  |  |  |  |  | 0.011 | (-0.032,0.053) |  | ^d^ | 0.010 | (-0.036,0.055) |  | ^d^ | |
| PFOA | | -0.061 | (-0.287,0.165) |  | | ^e^ | | -0.035 | (-0.263,0.194) |  | ^e^ |  |  |  |  |  |  |  |  | 0.010 | (-0.041,0.061) |  | ^f^ | 0.017 | (-0.034,0.067) |  | ^f^ | |
| PFOA (T2 vs. T1) | |  |  |  | |  | |  |  |  |  | -0.071 | (-0.109,-0.033) | *** | ^f^ | -0.068 | (-0.107,-0.029) | *** | ^f^ |  |  |  |  |  |  |  |  | |
| PFOA (T3 vs. T1) | |  |  |  | |  | |  |  |  |  | -0.039 | (-0.078,0.000) | * | ^f^ | -0.037 | (-0.077,0.003) | . | ^f^ |  |  |  |  |  |  |  |  | |
| PFNA | | -0.015 | (-0.178,0.149) |  | |  | | -0.009 | (-0.173,0.156) |  |  | -0.007 | (-0.046,0.032) |  | ^f^ | -0.007 | (-0.046,0.033) |  | ^f^ | 0.006 | (-0.030,0.042) |  | ^f^ | 0.007 | (-0.029,0.043) |  | ^f^ | |
| PFUDA | | 0.035 | (-0.117,0.188) |  | |  | | 0.059 | (-0.096,0.214) |  |  | 0.003 | (-0.033,0.039) |  | ^f^ | 0.001 | (-0.037,0.038) |  | ^f^ | 0.008 | (-0.026,0.041) |  | ^f^ | 0.007 | (-0.027,0.041) |  | ^f^ | |
| PFDA | | 0.045 | (-0.149,0.239) |  | |  | | 0.055 | (-0.139,0.248) |  |  | -0.029 | (-0.075,0.016) |  | ^f^ | -0.030 | (-0.076,0.015) |  | ^f^ | 0.004 | (-0.038,0.045) |  | ^f^ | 0.007 | (-0.034,0.048) |  | ^f^ | |
| PFHxS | | -0.147 | (-0.299,0.005) | . | |  | | -0.114 | (-0.271,0.043) |  |  | -0.022 | (-0.059,0.014) |  | ^f^ | -0.020 | (-0.058,0.018) |  | ^f^ | 0.024 | (-0.009,0.058) |  | ^f^ | 0.026 | (-0.008,0.060) |  | ^f^ | |
| PFOS | | -0.110 | (-0.274,0.054) |  | |  | | -0.061 | (-0.231,0.109) |  |  | 0.002 | (-0.038,0.041) |  | ^f^ | 0.009 | (-0.032,0.050) |  | ^f^ |  |  |  |  |  |  |  |  | |
| PFOS (T2 vs. T1) | |  |  |  | |  | |  |  |  |  |  |  |  |  |  |  |  |  | 0.014 | (-0.022,0.050) |  | ^f^ | 0.016 | (-0.021,0.052) |  | ^f^ | |
| PFOS (T3 vs. T1) | |  |  |  | |  | |  |  |  |  |  |  |  |  |  |  |  |  | 0.015 | (-0.023,0.052) |  | ^f^ | 0.008 | (-0.030,0.046) |  | ^f^ | |
| *All models adjusted for parental history of thyroid disease, season and hour of blood drawing* | | | | | | | | | | | | | | | | | | | | | | | | | | |  |  |
| *a Models further adjusted for total lipids (g/L), b Models further adjusted for total lipids (g/L), father's education level (< 12 years, 12 years, > 12 years), c Models further adjusted for total lipids (g/L), father's education level (< 12 years, 12 years, > 12 years) and passive tobacco smoking (yes/no), d Models further adjusted for total lipids (g/L), breastfeeding (none, ≤ 3 months, > 3 months), e Models further adjusted for father's education level (< 12 years, 12 years, > 12 years) and passive tobacco smoking (yes/no), f Models further adjusted for breastfeeding (none, ≤ 3 months, > 3 months), ⱡ Models adjusted for 12-year-old exposure, ⱡⱡ Models adjusted for 12-year-old POP serum concentration and POP cord-serum concentration, p,p'-DDE: dichlorodiphenyldichloroethylene; beta-HCH: beta hexachlorocyclohexane; PFOA: perfluorooctanoic acid; PFNA: perfluorononanoic acid; PFDA: perfluorodecanoic acid; PFUdA: perfluoroundecanoic acid; PFHxS: perfluorohexane sulfonate; PFOS: perfluorooctane sulfonate* | | | | | | | | | | | | | | | | | | | | | | | | | | |  |  |

**Table S6. Associations between POP serum concentrations and TSH, free T3 and free T4 serum concentrations at age 12. Complete set of confounders. Complementary adjustment for BMI**

|  | **TSH (mIU log-transformed)** | | | | | |  |  | **free T3 (pg/mL log-transformed)** | | | | | |  |  | **free T4 (ng/dL log-transformed)** | | | | | |  |  |  |
| --- | --- | --- | --- | --- | --- | --- | --- | --- | --- | --- | --- | --- | --- | --- | --- | --- | --- | --- | --- | --- | --- | --- | --- | --- | --- |
|  | **Boys** |  |  |  | **Girls** |  |  |  | **Boys** |  |  |  | **Girls** |  |  |  | **Boys** |  |  |  | **Girls** |  |  |  |  |
| **Exposure (µg/L log-transformed)** | **β** | **95%CI** | **p** |  | **β** | **95%CI** | **p** |  | **β** | **95%CI** | **p** |  | **β** | **95%CI** | **p** |  | **β** | **95%CI** | **p** |  | **β** | **95%CI** | **p** |  |  |
| PCB118 | -0.066 | (-0.191,0.058) |  | ^a^ | 0.030 | (-0.082,0.142) |  | ^c^ |  |  |  |  | -0.015 | (-0.044,0.015) |  | ^d^ | -0.015 | (-0.048,0.018) |  | ^a^ | -0.005 | (-0.033,0.022) |  | ^d^ |  |
| PCB118 (T2 vs. T1) |  |  |  |  |  |  |  |  | -0.021 | (-0.054,0.011) |  | ^c^ |  |  |  |  |  |  |  |  |  |  |  |  |  |
| PCB118 (T3 vs. T1) |  |  |  |  |  |  |  |  | -0.023 | (-0.054,0.008) |  | ^c^ |  |  |  |  |  |  |  |  |  |  |  |  |  |
| PCB138 | -0.063 | (-0.184,0.058) |  | ^a^ |  |  |  |  | -0.003 | (-0.029,0.023) |  | ^c^ | -0.025 | (-0.056,0.007) |  | ^d^ | -0.015 | (-0.047,0.017) |  | ^a^ | -0.003 | (-0.032,0.027) |  | ^d^ |  |
| PCB138 (T2 vs. T1) |  |  |  |  | -0.100 | (-0.251,0.050) |  | ^c^ |  |  |  |  |  |  |  |  |  |  |  |  |  |  |  |  |  |
| PCB138 (T3 vs. T1) |  |  |  |  | -0.005 | (-0.167,0.157) |  | ^c^ |  |  |  |  |  |  |  |  |  |  |  |  |  |  |  |  |  |
| PCB153 | -0.039 | (-0.145,0.068) |  | ^a^ | 0.037 | (-0.063,0.137) |  | ^c^ |  |  |  |  | -0.019 | (-0.050,0.013) |  | ^d^ | -0.010 | (-0.038,0.018) |  | ^a^ | -0.007 | (-0.036,0.022) |  | ^d^ |  |
| PCB153 (T2 vs. T1) |  |  |  |  |  |  |  |  | -0.017 | (-0.049,0.014) |  | ^c^ |  |  |  |  |  |  |  |  |  |  |  |  |  |
| PCB153 (T3 vs. T1) |  |  |  |  |  |  |  |  | 0.002 | (-0.031,0.034) |  | ^c^ |  |  |  |  |  |  |  |  |  |  |  |  |  |
| PCB180 | -0.013 | (-0.098,0.071) |  | ^a^ | 0.034 | (-0.045,0.114) |  | ^c^ |  |  |  |  | -0.018 | (-0.043,0.008) |  | ^d^ |  |  |  |  | -0.009 | (-0.033,0.015) |  | ^d^ |  |
| PCB180 (T2 vs. T1) |  |  |  |  |  |  |  |  | -0.018 | (-0.050,0.014) |  | ^c^ |  |  |  |  | -0.048 | (-0.087,-0.008) | * | ^a^ |  |  |  |  |  |
| PCB180 (T3 vs. T1) |  |  |  |  |  |  |  |  | -0.008 | (-0.043,0.026) |  | ^c^ |  |  |  |  | -0.015 | (-0.056,0.027) |  |  |  |  |  |  |  |
| Hexachlorobenzene | -0.131 | (-0.380,0.118) |  | ^a^ | 0.012 | (-0.255,0.279) |  | ^c^ | -0.042 | (-0.095,0.012) |  | ^b^ | -0.063 | (-0.137,0.012) | . | ^d^ | -0.059 | (-0.125,0.007) | . |  | 0.014 | (-0.055,0.084) |  | ^d^ |  |
| p,p'-DDE |  |  |  |  |  |  |  |  |  |  |  |  | -0.024 | (-0.058,0.009) |  | ^d^ | -0.002 | (-0.032,0.028) |  |  | 0.007 | (-0.024,0.038) |  | ^d^ |  |
| p,p'-DDE (T2 vs. T1) | -0.010 | (-0.156,0.135) |  | ^a^ | -0.003 | (-0.161,0.155) |  | ^c^ | 0.014 | (-0.017,0.045) |  | ^b^ |  |  |  |  |  |  |  |  |  |  |  |  |  |
| p,p'-DDE (T3 vs. T1) | -0.056 | (-0.218,0.106) |  | ^a^ | -0.023 | (-0.185,0.140) |  | ^c^ | -0.001 | (-0.035,0.034) |  | ^b^ |  |  |  |  |  |  |  |  |  |  |  |  |  |
| beta-HCH | -0.083 | (-0.186,0.020) |  | ^a^ | 0.115 | (0.007,0.223) | * | ^c^ | -0.004 | (-0.026,0.018) |  | ^b^ | -0.010 | (-0.040,0.021) |  | ^d^ |  |  |  |  |  |  |  |  |  |
| beta-HCH (T2 vs. T1) |  |  |  |  |  |  |  |  |  |  |  |  |  |  |  |  | -0.011 | (-0.049,0.027) |  | ^a^ | -0.016 | (-0.054,0.021) |  | ^d^ |  |
| beta-HCH (T3 vs. T1) |  |  |  |  |  |  |  |  |  |  |  |  |  |  |  |  | 0.019 | (-0.023,0.060) |  | ^a^ | 0.010 | (-0.031,0.052) |  | ^d^ |  |
| PFOA | 0.001 | (-0.175,0.177) |  |  | -0.067 | (-0.279,0.145) |  | ^e^ | -0.015 | (-0.053,0.023) |  | ^e^ |  |  |  |  |  |  |  |  | 0.004 | (-0.047,0.056) |  | ^f^ |  |
| PFOA (T2 vs. T1) |  |  |  |  |  |  |  |  |  |  |  |  | -0.061 | (-0.099,-0.024) | ** | ^f^ | 0.010 | (-0.028,0.048) |  |  |  |  |  |  |  |
| PFOA (T3 vs. T1) |  |  |  |  |  |  |  |  |  |  |  |  | -0.041 | (-0.081,-0.001) | * | ^f^ | 0.021 | (-0.018,0.060) |  |  |  |  |  |  |  |
| PFNA | 0.074 | (-0.089,0.236) |  |  | -0.037 | (-0.183,0.109) |  |  | -0.007 | (-0.043,0.029) |  | ^e^ | -0.011 | (-0.048,0.027) |  | ^f^ | 0.021 | (-0.025,0.067) |  |  | 0.004 | (-0.030,0.038) |  | ^f^ |  |
| PFUDA | -0.036 | (-0.150,0.078) |  |  | 0.040 | (-0.100,0.180) |  |  |  |  |  |  | 0.008 | (-0.028,0.044) |  | ^f^ | -0.003 | (-0.033,0.027) |  |  | 0.001 | (-0.031,0.034) |  | ^f^ |  |
|  |  |  |  |  |  |  |  |  | 0.005 | (-0.025,0.036) |  | ^e^ |  |  |  |  |  |  |  |  |  |  |  |  |  |
|  |  |  |  |  |  |  |  |  | -0.003 | (-0.042,0.036) |  | ^e^ |  |  |  |  |  |  |  |  |  |  |  |  |  |
| PFDA | -0.102 | (-0.238,0.033) |  |  | 0.031 | (-0.149,0.210) |  |  | -0.030 | (-0.059,-0.001) | * | ^e^ | -0.020 | (-0.065,0.026) |  | ^f^ | 0.009 | (-0.028,0.046) |  |  | 0.001 | (-0.041,0.042) |  | ^f^ |  |
|  |  |  |  |  |  |  |  |  |  |  |  |  |  |  |  |  |  |  |  |  |  |  |  |  |  |
|  |  |  |  |  |  |  |  |  |  |  |  |  |  |  |  |  |  |  |  |  |  |  |  |  |  |
| PFHxS | 0.057 | (-0.086,0.200) |  |  | -0.127 | (-0.271,0.017) | . |  | -0.011 | (-0.042,0.020) |  | ^e^ | -0.017 | (-0.054,0.020) |  | ^f^ |  |  |  |  | 0.018 | (-0.016,0.051) |  | ^f^ |  |
| PFHxS (T2 vs. T1) |  |  |  |  |  |  |  |  |  |  |  |  |  |  |  |  | 0.043 | (0.004,0.082) | * |  |  |  |  |  |  |
| PFHxS (T3 vs. T1) |  |  |  |  |  |  |  |  |  |  |  |  |  |  |  |  | 0.020 | (-0.018,0.059) |  |  |  |  |  |  |  |
| PFOS | -0.043 | (-0.163,0.077) |  |  | -0.099 | (-0.254,0.056) |  |  | -0.012 | (-0.038,0.014) |  | ^e^ | 0.003 | (-0.037,0.043) |  | ^f^ | 0.000 | (-0.032,0.031) |  |  |  |  |  |  |  |
| PFOS (T2 vs. T1) |  |  |  |  |  |  |  |  |  |  |  |  |  |  |  |  |  |  |  |  | -0.005 | (-0.040,0.030) |  | ^f^ |  |
| PFOS (T3 vs. T1) |  |  |  |  |  |  |  |  |  |  |  |  |  |  |  |  |  |  |  |  | 0.002 | (-0.035,0.040) |  | ^f^ |  |
| *All models adjusted for parental history of thyroid disease, season and hour of blood drawing* | | | | | | | | | | | | | | | | | | | | | | | | | |
| *a Models further adjusted for total lipids (g/L), b Models further adjusted for total lipids (g/L), father's education level (< 12 years, 12 years, > 12 years), c Models further adjusted for total lipids (g/L), father's education level (< 12 years, 12 years, > 12 years) and passive tobacco smoking (yes/no), d Models further adjusted for total lipids (g/L), breastfeeding (none, ≤ 3 months, > 3 months), e Models further adjusted for father's education level (< 12 years, 12 years, > 12 years) and passive tobacco smoking (yes/no), f Models further adjusted for breastfeeding (none, ≤ 3 months, > 3 months),* *p,p'-DDE: dichlorodiphenyldichloroethylene; beta-HCH: beta hexachlorocyclohexane; PFOA: perfluorooctanoic acid; PFNA: perfluorononanoic acid; PFDA: perfluorodecanoic acid; PFUdA: perfluoroundecanoic acid; PFHxS: perfluorohexane sulfonate; PFOS: perfluorooctane sulfonate* | | | | | | | | | | | | | | | | | | | | | | | | | |

**Table S7.1. Associations between POP serum concentrations and TSH, free T3 and free T4 serum concentrations at age 12. Minimal set of confounders. Stratified on pubertal stages – Girls**

|  | **TSH (mIU log-transformed)** | | | | |  |  |  |  |  |
| --- | --- | --- | --- | --- | --- | --- | --- | --- | --- | --- |
|  | **Tanner 1-2 for breast** | | | **Tanner 3 for breast** | | | **Tanner 4-5 for breast** | |  |  |
|  | **(n=54)** | |  | **(n=122)** | |  | **(n=51)** |  |  |  |
| **Exposure (µg/L log-transformed)** | **β** | **95%CI** | **p** | **β** | **95%CI** | **p** | **β** | **95%CI** | **p** | **p int** |
| PCB118 | -0.054 | (-0.310,0.201) |  | 0.013 | (-0.136,0.163) |  | -0.115 | (-0.370,0.141) |  |  |
| PCB138 (T2 vs. T1) | -0.303 | (-0.744,0.139) |  | -0.193 | (-0.411,0.024) | . | 0.120 | (-0.148,0.387) |  |  |
| PCB138 (T3 vs. T1) | -0.116 | (-0.498,0.266) |  | -0.110 | (-0.342,0.121) |  | -0.041 | (-0.340,0.259) |  |  |
| T12_BPC153_log | -0.003 | (-0.211,0.205) |  | -0.068 | (-0.204,0.069) |  | 0.010 | (-0.185,0.205) |  |  |
| T12_BPC180_log | 0.002 | (-0.162,0.166) |  | -0.058 | (-0.168,0.052) |  | 0.032 | (-0.133,0.197) |  |  |
| Hexachlorobenzene | -0.263 | (-0.721,0.194) |  | -0.202 | (-0.514,0.109) |  | 0.113 | (-0.387,0.614) |  |  |
| p,p'-DDE (T2 vs. T1) | -0.088 | (-0.525,0.349) |  | -0.095 | (-0.306,0.115) |  | -0.103 | (-0.376,0.170) |  | # |
| p,p'-DDE (T3 vs. T1) | 0.085 | (-0.285,0.455) |  | -0.249 | (-0.471,-0.026) | * | -0.094 | (-0.405,0.218) |  |  |
| beta-HCH | 0.034 | (-0.226,0.295) |  | 0.084 | (-0.078,0.245) |  | 0.039 | (-0.151,0.229) |  |  |
| PFOA | -0.023 | (-0.533,0.488) |  | -0.112 | (-0.454,0.230) |  | -0.280 | (-0.615,0.055) | . |  |
| PFNA | -0.036 | (-0.392,0.321) |  | 0.020 | (-0.182,0.222) |  | -0.196 | (-0.497,0.106) |  |  |
| PFUDA | 0.109 | (-0.215,0.434) |  | -0.039 | (-0.239,0.162) |  | -0.004 | (-0.265,0.257) |  |  |
| PFDA | 0.041 | (-0.359,0.441) |  | -0.094 | (-0.371,0.183) |  | 0.031 | (-0.309,0.370) |  |  |
| PFHxS | -0.290 | (-0.742,0.161) |  | -0.160 | (-0.351,0.031) | . | -0.101 | (-0.349,0.147) |  |  |
| PFOS | -0.001 | (-0.392,0.391) |  | -0.195 | (-0.406,0.016) | . | -0.080 | (-0.372,0.212) |  |  |

*All models adjusted for parental history of thyroid disease, season and hour of blood drawing; and further*

*adjusted for total lipids for PCBs, hexachlorobenzene, p,p'-DDE and beta-HCH. p int: p-value for interaction term between exposure and Tanner stage.*

*# p<0.2; ٠p<0.1; * p<0.05; ** p<0.01*

*p,p'-DDE: dichlorodiphenyldichloroethylene; beta-HCH: beta hexachlorocyclohexane; PFOA: perfluorooctanoic acid;*

*PFNA: perfluorononanoic acid; PFDA: perfluorodecanoic acid; PFUdA: perfluoroundecanoic acid;*

*PFHxS: perfluorohexane sulfonate; PFOS: perfluorooctane sulfonate*

**Table S7.2. Associations between POP serum concentrations and TSH, free T3 and free T4 serum concentrations at age 12. Minimal set of confounders. Stratified on pubertal stages – Girls**

|  | **free T3 (pg/mL log-transformed)** | | | | |  |  |  |  |  | | **free T4 (ng/dL log-transformed)** | | | | |  | |  |  |  |  |
| --- | --- | --- | --- | --- | --- | --- | --- | --- | --- | --- | --- | --- | --- | --- | --- | --- | --- | --- | --- | --- | --- | --- |
|  | **Tanner 1-2 for breast** | | | **Tanner 3 for breast** | |  | **Tanner 4-5 for breast** | | |  | **Tanner 1-2 for breast** | | |  | **Tanner 3 for breast** | | | | **Tanner 4-5 for breast** | |  |  |
|  | **(n=54)** | |  | **(n=122)** | |  | **(n=51)** |  |  |  | | **(n=54)** |  |  | **(n=122)** | | |  | **(n=51)** |  |  |  |
| **Exposure (µg/L log-transformed)** | **β** | **95%CI** |  | **β** | **95%CI** |  | **β** | **95%CI** |  | **p int** | | **β** | **95%CI** |  | **β** | **95%CI** | |  | **β** | **95%CI** |  | **p int** |
| PCB118 | -0.016 | (-0.065,0.032) |  | -0.018 | (-0.054,0.019) |  | 0.029 | (-0.069,0.126) |  |  | | 0.013 | (-0.056,0.082) |  | 0.008 | (-0.021,0.038) | |  | 0.009 | (-0.063,0.082) |  |  |
| PCB138 | -0.013 | (-0.057,0.031) |  | -0.018 | (-0.055,0.019) |  | 0.025 | (-0.054,0.104) |  |  | | 0.006 | (-0.056,0.069) |  | 0.024 | (-0.006,0.054) | |  | -0.001 | (-0.060,0.058) |  |  |
| PCB153 | -0.006 | (-0.045,0.034) |  | -0.010 | (-0.044,0.023) |  | 0.036 | (-0.037,0.109) |  |  | | 0.004 | (-0.052,0.060) |  | 0.023 | (-0.004,0.050) | | . | -0.006 | (-0.061,0.048) |  |  |
| PCB180 | -0.002 | (-0.034,0.029) |  | -0.006 | (-0.033,0.021) |  | 0.030 | (-0.032,0.091) |  |  | | 0.000 | (-0.044,0.044) |  | 0.020 | (-0.002,0.041) | | . | -0.010 | (-0.056,0.036) |  |  |
| Hexachlorobenzene | -0.027 | (-0.116,0.061) |  | -0.047 | (-0.123,0.029) |  | 0.036 | (-0.153,0.226) |  |  | | 0.073 | (-0.049,0.196) |  | 0.050 | (-0.012,0.112) | |  | 0.050 | (-0.089,0.189) |  |  |
| p,p'-DDE | -0.010 | (-0.061,0.042) |  | -0.028 | (-0.067,0.011) |  | 0.033 | (-0.051,0.118) |  | # | | 0.039 | (-0.032,0.110) |  | 0.011 | (-0.021,0.043) | |  | 0.017 | (-0.046,0.080) |  |  |
| beta-HCH | -0.006 | (-0.056,0.044) |  | 0.009 | (-0.030,0.049) |  | 0.011 | (-0.061,0.082) |  |  | |  |  |  |  |  | |  |  |  |  |  |
| beta-HCH (T2 vs. T1) |  |  |  |  |  |  |  |  |  |  | | 0.031 | (-0.071,0.134) |  | -0.003 | (-0.047,0.042) | |  | -0.010 | (-0.087,0.067) |  |  |
| beta-HCH (T3 vs. T1) |  |  |  |  |  |  |  |  |  |  | | 0.088 | (-0.016,0.191) | . | 0.007 | (-0.042,0.055) | |  | 0.030 | (-0.058,0.117) |  |  |
| PFOA |  |  |  |  |  |  |  |  |  |  | | 0.091 | (-0.039,0.220) |  | -0.001 | (-0.071,0.068) | |  | 0.005 | (-0.092,0.103) |  |  |
| PFOA (T2 vs. T1) | 0.003 | (-0.086,0.091) |  | -0.098 | (-0.147,-0.048) | *** | -0.078 | (-0.173,0.017) |  | # | |  |  |  |  |  | |  |  |  |  |  |
| PFOA (T3 vs. T1) | -0.023 | (-0.107,0.062) |  | -0.052 | (-0.105,0.001) | . | -0.028 | (-0.143,0.088) |  |  | |  |  |  |  |  | |  |  |  |  |  |
| PFNA | 0.013 | (-0.061,0.088) |  | -0.023 | (-0.073,0.026) |  | 0.002 | (-0.113,0.117) |  |  | | 0.021 | (-0.071,0.113) |  | -0.001 | (-0.042,0.040) | |  | 0.039 | (-0.047,0.125) |  |  |
| PFUDA | 0.009 | (-0.059,0.077) |  | -0.013 | (-0.061,0.036) |  | 0.078 | (-0.016,0.173) |  |  | | -0.015 | (-0.100,0.069) |  | 0.004 | (-0.037,0.045) | |  | 0.060 | (-0.011,0.132) | . |  |
| PFDA | -0.020 | (-0.103,0.064) |  | -0.034 | (-0.101,0.034) |  | 0.016 | (-0.110,0.143) |  |  | | 0.015 | (-0.088,0.119) |  | 0.002 | (-0.054,0.059) | |  | 0.045 | (-0.050,0.139) |  |  |
| PFHxS | -0.040 | (-0.135,0.055) |  | -0.023 | (-0.070,0.024) |  | 0.030 | (-0.063,0.123) |  |  | | 0.110 | (-0.005,0.224) | . | -0.001 | (-0.040,0.039) | |  | 0.053 | (-0.015,0.122) |  | . |
| PFOS | 0.045 | (-0.036,0.126) |  | -0.010 | (-0.062,0.042) |  | -0.011 | (-0.121,0.098) |  |  | |  |  |  |  |  | |  |  |  |  |  |
| PFOS (T2 vs. T1) |  |  |  |  |  |  |  |  |  |  | | 0.061 | (-0.042,0.165) |  | -0.014 | (-0.058,0.030) | |  | -0.001 | (-0.070,0.067) |  |  |
| PFOS (T3 vs. T1) |  |  |  |  |  |  |  |  |  |  | | 0.058 | (-0.042,0.158) |  | -0.011 | (-0.055,0.033) | |  | 0.064 | (-0.029,0.157) |  |  |

*All models adjusted for parental history of thyroid disease, season and hour of blood drawing; and further*

*adjusted for total lipids for PCBs, hexachlorobenzene, p,p'-DDE and beta-HCH. p int: p-value for interaction term between exposure and Tanner stage.*

*# p<0.2; ٠p<0.1; * p<0.05; ** p<0.01*

*p,p'-DDE: dichlorodiphenyldichloroethylene; beta-HCH: beta hexachlorocyclohexane; PFOA: perfluorooctanoic acid;*

*PFNA: perfluorononanoic acid; PFDA: perfluorodecanoic acid; PFUdA: perfluoroundecanoic acid;*

*PFHxS: perfluorohexane sulfonate; PFOS: perfluorooctane sulfonate*
